# Causes of endemic radiation in the Caribbean: evidence from the historical biogeography and diversification of the butterfly genus *Calisto* (Nymphalidae: Satyrinae: Satyrini)

**DOI:** 10.1186/s12862-014-0199-7

**Published:** 2014-09-16

**Authors:** Pável Matos-Maraví, Rayner Núñez Águila, Carlos Peña, Jacqueline Y Miller, Andrei Sourakov, Niklas Wahlberg

**Affiliations:** Laboratory of Genetics, Department of Biology, University of Turku, FI-20014 Turku, Finland; School of Biological Sciences, University of South Bohemia and Institute of Entomology, Biology Centre AS CR, CZ-37005 Ceske Budejovice, Czech Republic; División de Colecciones Zoológicas y Sistemática, Instituto de Ecología y Sistemática, Carretera de Varona km 3.5, Capdevila, Boyeros Ciudad de La Habana, Cuba; McGuire Center for Lepidoptera and Biodiversity, Florida Museum of Natural History, University of Florida, Gainesville, FL 32611 USA

**Keywords:** Caribbean, Ecological limits, Historical biogeography, Intra-island diversification, Island-island vicariance, Lepidoptera, Molecular phylogeny

## Abstract

**Background:**

*Calisto* is the largest butterfly genus in the West Indies but its systematics, historical biogeography and the causes of its diversification have not been previously rigorously evaluated. Several studies attempting to explain the wide-ranging diversity of *Calisto* gave different weights to vicariance, dispersal and adaptive radiation. We utilized molecular phylogenetic approaches and secondary calibrations points to estimate lineage ages. In addition, we used the dispersal-extinction-cladogenesis model and Caribbean paleogeographical information to reconstruct ancestral geographical distributions. We also evaluated different models of diversification to estimate the dynamics of lineage radiation within *Calisto*. By understanding the evolution of *Calisto* butterflies, we attempt to identify the main processes acting on insular insect diversity and the causes of its origin and its maintenance.

**Results:**

The crown age of *Calisto* was estimated to the early Oligocene (31 ± 5 Ma), and a single shift in diversification rate following a diversity-dependent speciation process was the best explanation for the present-day diversity found within the genus. A major increase in diversification rate was recovered at 14 Ma, following geological arrangements that favoured the availability of empty niches. Inferred ancestral distributional ranges suggested that the origin of extant *Calisto* is in agreement with a vicariant model and the origin of the Cuban lineage was likely the result of vicariance caused by the Cuba-Hispaniola split. A long-distance dispersal was the best explanation for the colonization of Jamaica and the Bahamas.

**Conclusions:**

The ancestral geographical distribution of *Calisto* is in line with the paleogeographical model of Caribbean colonization, which favours island-to-island vicariance. Because the sister lineage of *Calisto* remains ambiguous, its arrival to the West Indies remains to be explained, although, given its age and historical biogeography, the hypothesized GAARlandia land bridge might have been a plausible introduction route from continental America. Intra-island radiation caused by ecological innovation and the abiotic creation of niche spaces was found to be the main force shaping *Calisto* diversity and island endemism in Hispaniola and Cuba.

**Electronic supplementary material:**

The online version of this article (doi:10.1186/s12862-014-0199-7) contains supplementary material, which is available to authorized users.

## Background

The Caribbean has been an important model system for studying biotic over-water dispersal from continents and island colonization [1-4], as well as vicariance [5,6] as mechanisms for the origin of diversity, and within-island diversification as mediators of species richness and endemism [7,8]. The geological evolution of the region has certainly had a strong influence on the diversification of species there, and a general understanding of the former is crucial to an understanding of the latter.

The larger islands of the Greater Antilles (i.e. Cuba, Hispaniola, Jamaica and Puerto Rico) were repeatedly submerged until the mid/late Eocene (~40 Ma) [3,6]. A general terrene uplift is likely to have occurred during the mid-Eocene and the early Oligocene (~45-30 Ma), and some authors hypothesized the existence of a land corridor connecting northern South America to the Greater Antilles and subaerial Aves Ridge (GAARlandia, ~35-33 Ma) [6,9], although this is still under debate [10]. Hispaniola and Puerto Rico were physically connected until the formation of the Mona Passage, becoming fully separated during the late Oligocene to early Miocene (~30-20 Ma) [11,12]. Later, during the early to mid-Miocene, the aerial connection between eastern Cuba and northern Hispaniola was interrupted by the expansion of the Windward Passage (~17-14 Ma) [6,13].

Northern and southern Hispaniola paleoislands collided in the mid Miocene (*ca*. 15–10 Ma) [6,14,15], triggering the initial uplift of south-western Hispaniolan mountains as well as the significant elevation of the Cordillera Central [16,17]. Multiple marine incursions in the Cul-de-Sac/Enriquillo depression repeatedly separated northern and southern paleoislands until the Plio-Pleistocene (~2.5 Ma) [15,18]. Cuba was fragmented into distinct land blocks comprising the current western, central and eastern parts of the island until the late Miocene, when the closure of the Havana-Matanzas Channel began some 8–6 Ma [6]. On the other side, Jamaica was continuously submerged until *ca*. 12 Ma [19]. The western Jamaica land block was temporally aerial and connected to Central America during the early to mid-Eocene [6,20], whereas eastern Jamaica (Blue Mountains Block) was apparently connected to GAARlandia through the southern peninsula of Hispaniola during ~35-33 Ma [4,6]. Most Bahamian shallows and keys were repeatedly submerged during the Pliocene and Pleistocene (~4-0.5 Ma) [21].

The butterfly genus *Calisto* (Nymphalidae, Satyrinae, Satyrini) is the only satyrine group occurring in the Caribbean region [22,23]. This genus exhibits remarkable radiation and significantly contributes to the high butterfly endemism seen in the region [24,25]. The genus *Calisto* comprises 44 described species, all geographically restricted to single islands [23,26-29]; 11 distributed in Cuba, 1 in Puerto Rico, 1 in Anegada Island, 1 in Jamaica, 2 in the Bahamas and the remaining 28 species occurring in Hispaniola. Molecular data has given insight into the cryptic condition of several taxa in Hispaniola [27], as well as assisted in determining the phylogenetic relationships of Cuban taxa [28].

Even though the monophyly of the genus appears to be clear [27], its position within the taxonomic tribe Satyrini has not been resolved. Morphological studies classify *Calisto* within the subtribe Pronophilina [23], closely related to the Neotropical genus *Eretris* [30,31]. However, this has not been corroborated at the molecular level [32]. Certain morphological similarities have even led some authors to propose African affinities with, for instance, the subtribe Ypthimina (Satyrini) [33] and the satyrine tribe Dirini [34,35].

Regardless of the phylogenetic position of *Calisto*, a continental origin of the genus is the most plausible explanation, as no other extant satyrine butterflies with the potential of being a closely related group are found in the Greater Antilles; thus, its ancestors would have necessarily arrived to the Caribbean from the nearby American continent [31,33,36]. Once *Calisto* colonized the Greater Antilles, further differentiation by vicariance [31,37], within-island diversification [28,36] or adaptive radiation [27] might have shaped the evolution of these butterflies.

In this study, we aim to elucidate the phylogenetic affinities and to identify the main drivers of the diversification and distribution of *Calisto* by using a secondarily calibrated molecular phylogeny. We also aim to reconstruct the historical biogeography of *Calisto* and to evaluate possible changes in diversification rates throughout the evolution of the genus. Intra-island differentiation appears to be an important factor for the radiation of these butterflies, a phenomenon observed in other Caribbean animal lineages [2,4,9,38-40]. However, even though rapid diversification driven by ecological evolution is plausible explanation considering the diversity of Caribbean habitats, niche saturation and island size may have imposed diversification limits [38] which could have restricted the diversity and geographical distribution of *Calisto*.

## Results

### Systematics and divergence dates of *Calisto*

**Figure 1:**
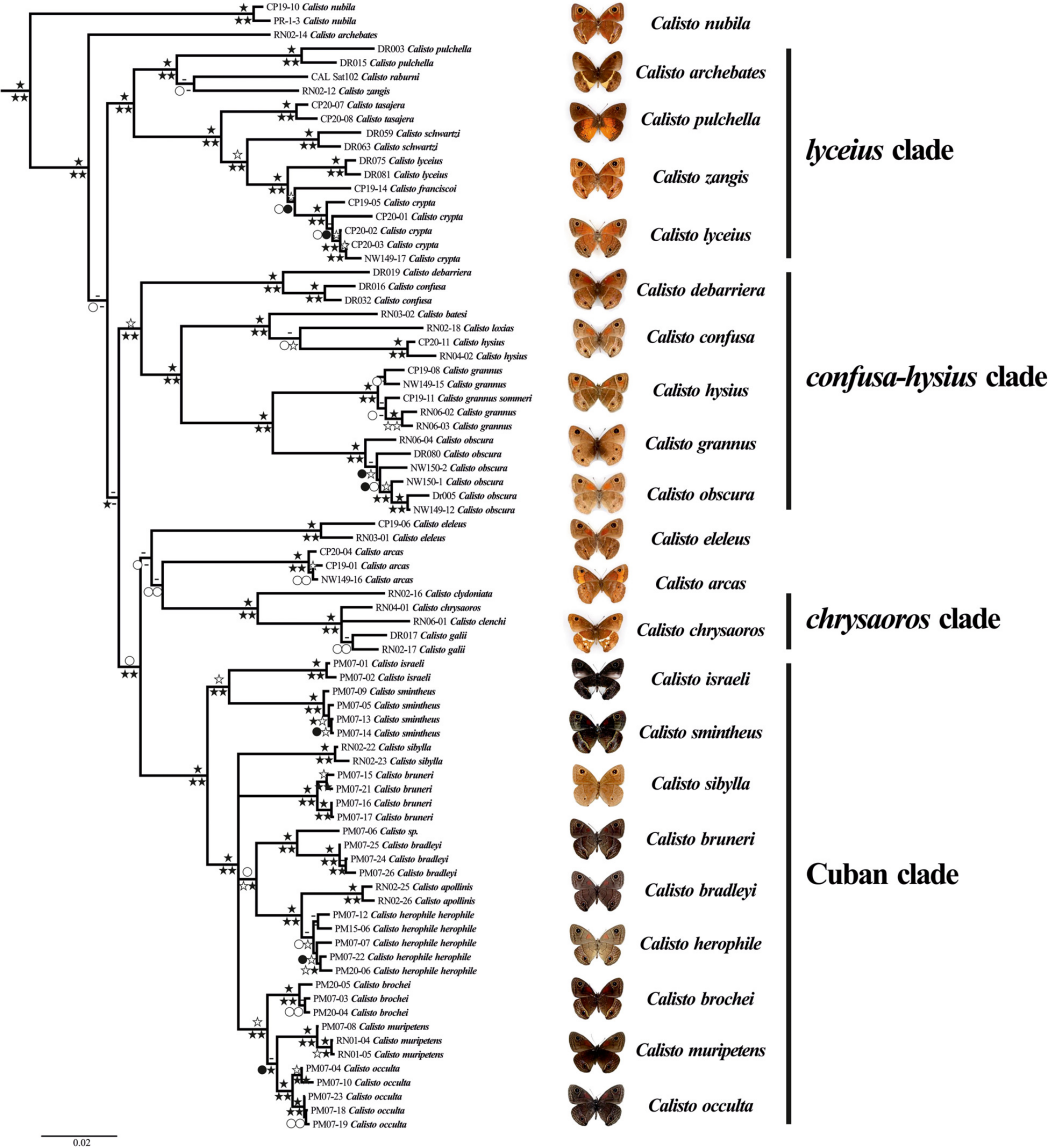
**BI consensus phylogeny using the combined dataset partitioned by gene.** Support values are represented by symbols on the left of each node, where the upper symbol is the bootstrap (BS) support value from the ML analysis, and the left bottom symbol is the posterior probability (PP) of the Bayesian Inference (BI) from the gene partition analysis and from the partition-by-bins analysis on the right. Filled stars are strong support values of 0.95-1.00 and 90–100 for PP and BS respectively, stars are 0.85-0.94 and 75–89, filled circles are 0.75-0.84 and 65–74 whereas circles are 0.50-0.74 and 50–64. Dashes (−) are unresolved nodes on each analysis. Branch lengths represent expected substitutions/site estimated in the BI analysis.

Our phylogenetic inferences using single gene datasets are congruent with the combined analyses, recovering the main clades within *Calisto* (Additional file 1). Moreover, the combined analyses were consistent regardless of the method used and the partitioning strategy (Figure 1). A summary of the dataset properties is presented in Table 1.

**Table 1:** **Partition strategies for phylogenetic analyses of the combined dataset**

**Partitions**	**Base pairs**	***Variable***	***Informative***	***Subs. model***	***m*** **(ratemult)**	***Alpha*** **(Γ shape)**	***Tree likelihood***
***Gene strategy***							
COI	1487	651	486	GTR + G	1.569	0.245	−18584.5
CAD	850	319	218	HKY + G	0.749	0.282	−4766.8
EF1a	1240	432	290	GTR + G	0.768	0.222	−21045.8
GAPDH	691	264	196	GTR + G	0.864	0.232	−13990.5
RPS5	617	228	183	GTR + G	0.694	0.198	−11920.2
WINGLESS	400	191	130	K80 + G	0.848	0.34	−8617.8
***“Bin” strategy***							
BIN1	2727	-	-	F81	0.0002	-	-
BIN2-BIN10	652	-	-	GTR	0.57	-	-
BIN11	1269	-	-	GTR + G	1.457	1.601	-
BIN12	637	-	-	GTR + G	4.828	4.389	-

Number of variable and phylogenetically informative sites in our *Calisto* data are shown by gene partition. Substitution model was selected based on BIC calculations in jModelTest [41]. Rate multiplier (*m*) and Gamma-shape (*alpha*) parameters are from BI whereas the *tree likelihood* for each gene partition are from the dating analysis using normal distribution for the calibration points and the birth-death process. Other dating analyses have similar values as shown in *tree likelihood*.

**Figure 2:**
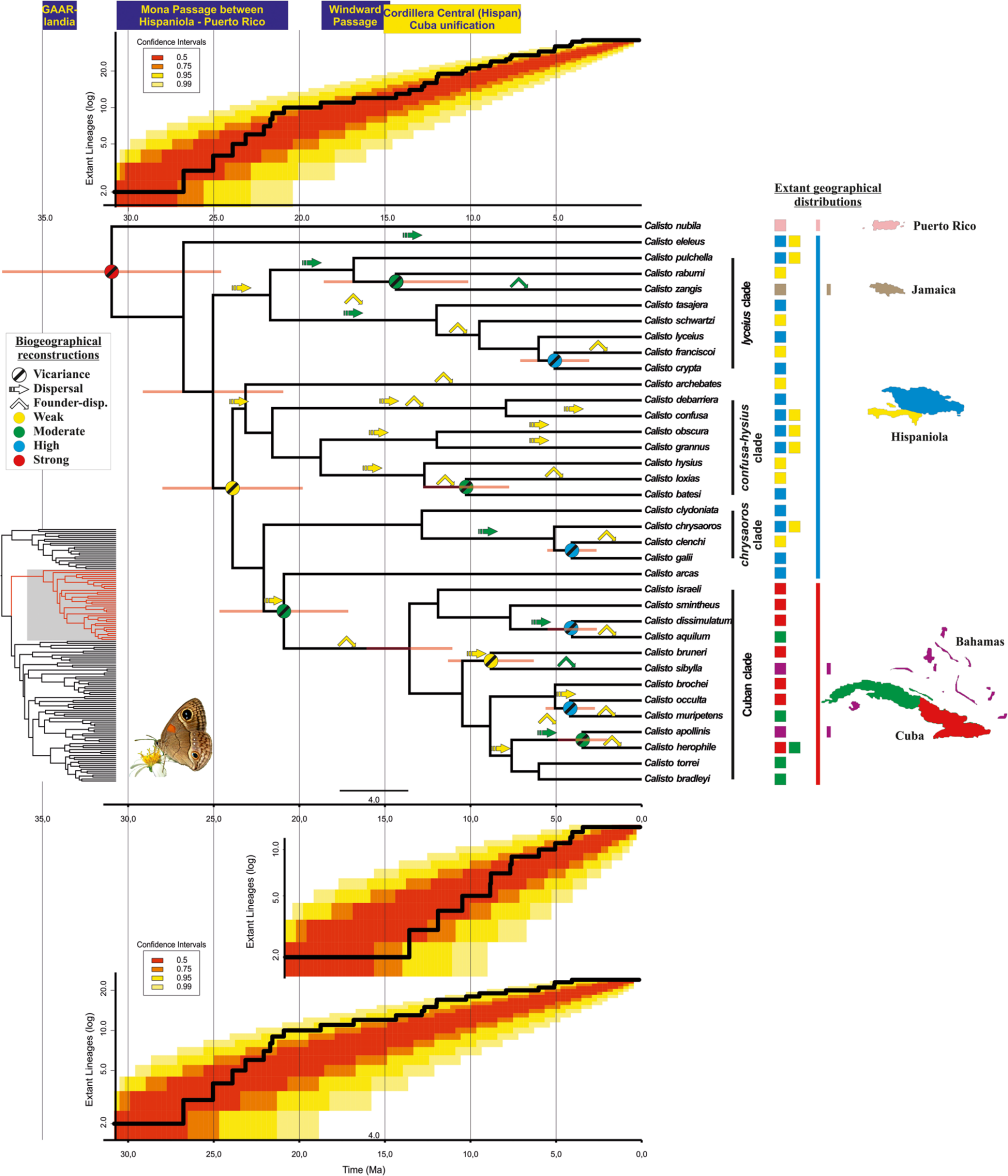
**Dated phylogram and a consensus biogeographical history.** The ultrametric tree is scaled in Ma. Symbols on each critical node/branch are depicted as the most likely scenarios: vicariance, dispersal or founder-event. Colours on each symbol represent the level of support. Horizontal bars on nodes represent 95% credibility intervals. The phylogeny in the bottom left is the Satyrini tree, with the *Calisto* clade showing in red. Extant distributions of *Calisto*, following the subdivision of the Greater Antilles, are represented by coloured squares. The main geological events through time are depicted on top of the figure following the time scale in Ma. Lineage Through Time (LTT) plot of extant *Calisto* diversity (log scale) vs. time (Ma) is shown above the phylogeny, whereas the LTT of the Cuban clade is below the tree and the LTT of the Hispaniolan lineages is in the bottom of the figure. LTT plots follow the time scale of the phylogeny in Ma. Confidence intervals for LTT are displayed as coloured ranges.

*Calisto nubila* split early in the evolution of the genus, becoming an old and separate entity. The lineage did not apparently diversify further within Puerto Rico, although *C. anegadensis* on Anegada Island might have been derived from it based on morphological similarities [26]. Three main monophyletic groups from Hispaniola are identified: the *lyceius*-, the *confusa*-*hysius* and the *chrysaoros* clades. *Calisto zangis* from Jamaica is likely to have had Hispaniolan ancestors as it belongs to the “*lyceius* clade”. The monophyletic group consisting of Cuban and Bahamian *Calisto* is closely related to Hispaniolan lineages such as *C. arcas* and the “*chrysaoros* clade”, although the relationship among them was not resolved with strong support (Figures 1 and 2). A revised checklist of the genus *Calisto* is presented in Table 2.

**Table 2:** **Revised checklist of the genus** ***Calisto*** **(Lepidoptera: Nymphalidae: Satyrinae: Satyrini)**

**Island**	**Proposed species for** ***Calisto***	**Classification within** ***Calisto***
Anegada	*Calisto anegadensis*	Related to *Calisto nubila*. Reference [26]
Bahamas	*Calisto apollinis*	Cuban clade 2
Bahamas	*Calisto sibylla*	Cuban clade 2
Cuba	*Calisto aquilum*	Cuban clade 1
Cuba	*Calisto bradleyi*	Cuban clade 2
Cuba	*Calisto brochei*	Cuban clade 2
Cuba	*Calisto bruneri*	Cuban clade 2
Cuba	*Calisto dissimulatum*	Cuban clade 1
Cuba	*Calisto herophile*	Cuban clade 2
Cuba	*Calisto israeli*	Cuban clade 1
Cuba	*Calisto muripetens*	Cuban clade 2
Cuba	*Calisto occulta*	Cuban clade 2
Cuba	*Calisto smintheus*	Cuban clade 1
Cuba	*Calisto torrei*	Cuban clade 2
Hispaniola (North and South)	*Calisto chrysaoros*	*chrysaoros* clade
Hispaniola (North and South)	*Calisto confusa*	*confusa-hysius* clade
Hispaniola (North and South)	*Calisto eleleus*	*Incertae sedis*
Hispaniola (North and South)	*Calisto grannus*	*confusa-hysius* clade
Hispaniola (North and South)	*Calisto obscura*	*confusa-hysius* clade
Hispaniola (North and South)	*Calisto pulchella*	*lyceius* clade
Hispaniola (North)	*Calisto ainigma*	Related to *Calisto eleleus*. Reference [42]
Hispaniola (North)	*Calisto arcas*	*Incertae sedis*
Hispaniola (North)	*Calisto batesi*	*confusa-hysius* clade
Hispaniola (North)	*Calisto clydoniata*	*chrysaoros* clade
Hispaniola (North)	*Calisto crypta*	*lyceius* clade
Hispaniola (North)	*Calisto debarriera*	*confusa-hysius* clade
Hispaniola (North)	*Calisto galii*	*chrysaoros* clade
Hispaniola (North)	*Calisto lyceius*	*lyceius* clade
Hispaniola (North)	*Calisto neochma*	Related to *Calisto clydoniata*. Reference [43]
Hispaniola (North)	*Calisto tasajera*	*lyceius* clade
Hispaniola (North)	*Calisto wetherbeei*	Related to *Calisto archebates*. Reference [44]
Hispaniola (South)	*Calisto archebates*	*Incertae sedis*
Hispaniola (South)	*Calisto clenchi*	*chrysaoros* clade
Hispaniola (South)	*Calisto franciscoi*	*lyceius* clade
Hispaniola (South)	*Calisto hysius*	*confusa-hysius* clade
Hispaniola (South)	*Calisto loxias*	*confusa-hysius* clade
Hispaniola (South)	*Calisto pauli*	Related to *Calisto hysius* (*C. herophile & C. sibylla*?). Reference [45]
Hispaniola (South)	*Calisto raburni*	*lyceius* clade
Hispaniola (South)	*Calisto schwartzi*	*lyceius* clade
Hispaniola (South)	*Calisto thomasi*	Related to *Calisto confusa*. Reference [45]
Hispaniola (South)	*Calisto tragius*	Related to *Calisto eleleus*. Reference [46]
Hispaniola (South)	*Calisto woodsi*	Related to *Calisto pauli*. Reference [45]
Jamaica	*Calisto zangis*	*lyceius* clade
Puerto Rico	*Calisto nubila*	Puerto Rican lineage

Each island and its fauna is shown according to the phylogenetic relationships presented in this study. Hispaniola is subdivided in northern and southern paleoislands. The eight species that were not included in this work are listed with their putative sister taxa.

The genus *Calisto* was not recovered within any valid Satyrini subtribes. Instead, our BEAST reconstructions place it sister to all sampled subtribes except Euptychiina with low support values (posterior probability around 0.60-0.65) (Additional file 1). The exclusion of the genus *Euptychia* (which apparently caused long branch attraction in a different dataset [32]) only increases the support for such a placement to moderate values (around 0.80-0.85). Using birth-death/Yule and normal/uniform as tree processes and calibration distributions respectively does not result in any significant difference in both tree topology and estimated ages (Additional file 1, Figure 2). Height posterior distributions displayed normally whereas summarizing the trees as means or medians height showed no significant difference. The crown age of *Calisto* is inferred at 31 Ma (±5 Ma) in all cases except when Yule process and the calibration normal distribution are used together, in which case the estimate is at 33 Ma (±7 Ma).

### Historical biogeography reconstruction

**Figure 3:**
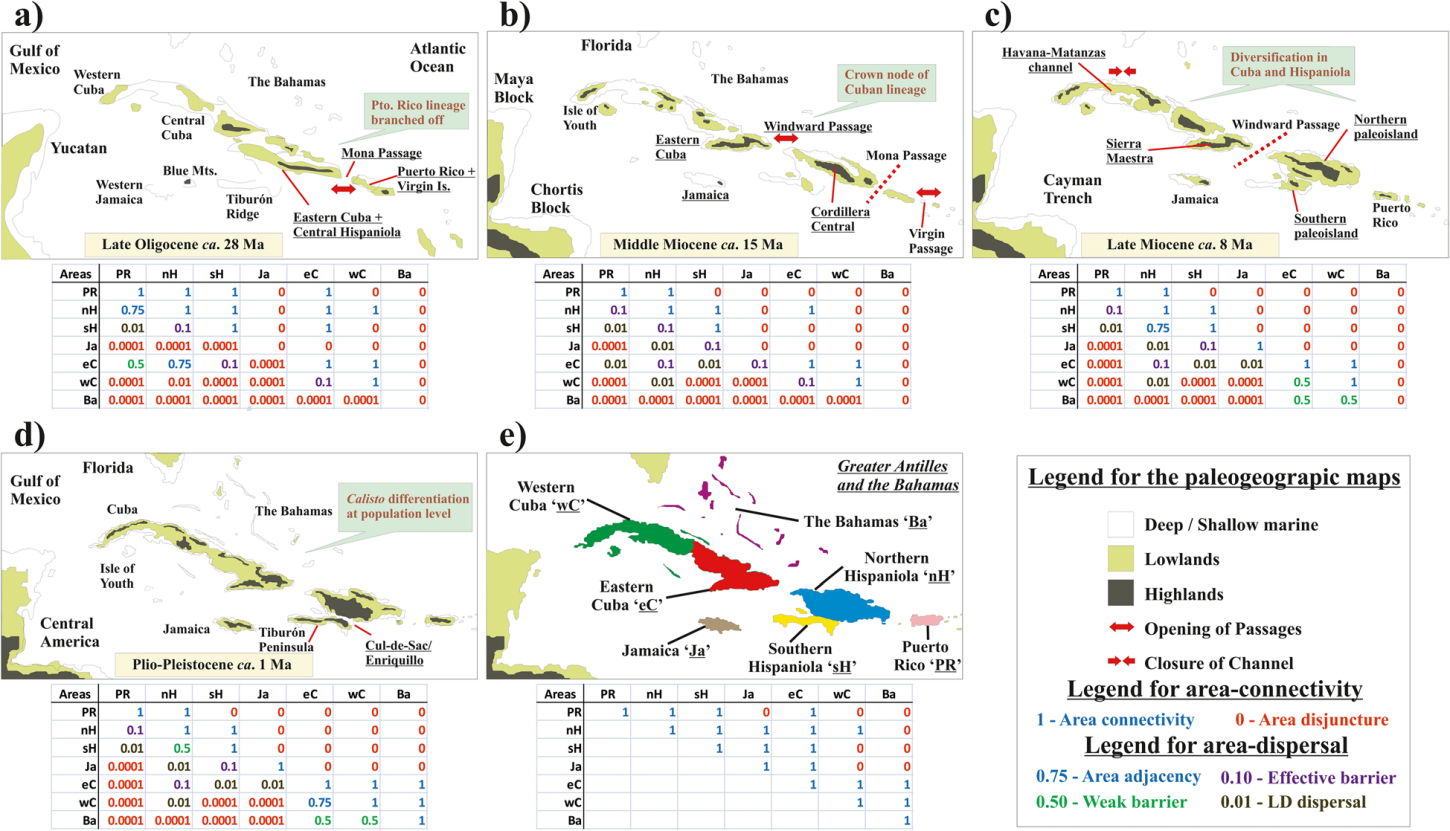
**Geological history of the Greater Antilles and Bahamas and the evolution of** ***Calisto.*** **a)** The crown node of extant *Calisto* occurred in the late Oligocene, and the split of Puerto Rico and Hispaniola coincided with the divergence of both faunas. **b)** In the middle Miocene, Hispaniola and Cuba were physically separated, promoting the isolation of lineages on both islands. **c)** The creation of new niche space in Hispaniola and Cuba triggered the radiation of *Calisto* by the mid/late Miocene; Cuban land blocks were unified and Hispaniolan mountain ranges were rapidly uplifted during the late Miocene. **d)** Temporal isolation/connection of areas within each island during the glacial/interglacial cycles of the Pleistocene. **e)** Present-day Greater Antilles coded and coloured as our biogeographical analyses. Maps were modified from [6]. Area connectivity and dispersal rates used in our biogeographical analyses are shown below each time period (a: 31–20 Ma, b: 10–20 Ma, c: 5–10 Ma, and d: 5 Ma to present). Upper-right of each table **(a-d)** are area-adjacency values as used in BioGeoBEARS and values in **(e)** were used in Lagrange C++. Dispersal probability, as used in TS analyses, are displayed below on each table. LD is long-distance dispersal including one extra area. Values of 0.0001 were assigned to LD involving more than one water barrier and extra areas.

There was no statistical difference in the global likelihood between the non-time-stratified analyses NS0 and NS1 (Table 3). Excluding unlikely area connections (NS1), resulted in a Puerto Rico-northern Hispaniola (PR-nH) distribution on the crown node of *Calisto*, whereas NS0 equally preferred PR along with both nH and sH (southern Hispaniola) (Table 4, Figure 3). Similarly, NS0 and NS1 were unable to discern between dispersal and vicariance for the origin of Cuban *Calisto*. The time-stratified analysis TS1 favoured vicariance over dispersal in all cases and TS2 inferred a PR-sH origin of *Calisto* and vicariance for the origin of Cuban diversity. However, TS2 analysis did not improve the global likelihood of the inference over TS1. Root optimizations significantly favoured a PR-sH distribution and vicariance as the cause of the Cuban clade split from its sister Hispaniolan lineages.

**Table 3:** **Estimated parameters and global likelihoods on each of the biogeographical analyses**

**Non-stratified**	**Global in-likelihood**	***d***	***e***	***j***	**Stratified**	**Global in-likelihood**	***D***	***e***	***j***
*Lagrange C++*					*Lagrange C++*				
*NS0	**−73.5273**	**0.2700**	**0.0102**	**-**	*TS1	**−88.2411**	**0.6363**	**0.0003**	**-**
*NS1	**−73.8920**	**0.3872**	**0.0146**	**-**	TS2	−93.1298	0.7224	0.0005	-
*Root optimization*					*Root optimization*				
*NS1_PR-sH	**−72.1124**	**0.4854**	**0.0053**	**-**	*TS2_PR-sH	**−94.3276**	**0.6718**	**0.0005**	**-**
NS1_PR-nH	−74.5323	0.3517	0.0114	-	TS2_PR-nH	−97.4394	0.6162	0.0025	-
NS1_PR-nH-eC	−76.5541	0.3825	0.0118	-	TS2_PR-nH-eC	−97.6103	0.6174	0.0017	-
NS1_PR-sH-eC	−76.6322	0.3313	0.0086	-	TS2_PR-nH-sH	−97.8598	0.5802	0.0019	-
NS1_PR-nH-sH	−77.1248	0.3048	0.0083	-	TS2_PR-sH-eC	−98.4153	0.5613	0.0001	-
NS1_nH	−78.4231	0.3522	0.0141	-	TS2_sH	−99.4647	0.7906	0.0050	-
NS1_PR	−80.0185	0.3219	0.0059	-	TS2_nH	−101.7850	0.6684	0.0089	-
NS1_sH	−80.2485	0.2874	0.0082	-	TpS2_PR	−103.8450	0.6690	0.0063	-
*BioGeoBEARS DEC model*					*BioGeoBEARS DEC model*				
*NS1- ***j***	**−78.7492**	**0.0020**	**0.0000**	**0.0709**	*TS1- ***j***	**−63.8944**	**0.0581**	**0.0042**	**0.5821**
NS1	−97.9848	0.0054	0.0069	-	TS1	−76.7373	0.1211	0.0098	-

The best models from each type of analyses are highlighted in bold text and marked with an asterisk (*). Parameter *d* is the rate of “dispersal” or range expansion, *e* is the rate of “extinction” or range contraction, and *j* is the relative weight of jump dispersal. *j* is cladogenetic, and *d* and *e* are anagenetic processes. Model-comparison between the BioGeoBEARS models resulted in Akaike weights favouring TS1-*j* with a relative probability of 0.999 of it being the best model. Similarly, LRT between TS1 and TS1-*j*, the two best models, rejected TS1 as the null model with fewer parameters with p-value of 4.02e^−07^.

**Table 4:** **Biogeographical reconstructions for the evolution of** ***Calisto***

		**Crown Calisto**	**Stem Cuban Calisto**	**Stem Jamaican Calisto**	**Stem Bahamian C. sibylla**	**Stem Bahamian C. apollinis**	**N° vicariant events**	**N° dispersal events**
Lagrange C++	NS0	PR-sH: 0.24 (-74.94);	nH-eC: 0.44 (-74.34);	*sH-Ja: 0.61 (-74.01);	eC-Ba: 0.55 (-74.12);	*eC-wC-Ba: 0.83 (-73.71)	8	5
		PR-nH-sH: 0.16 (-75.36);	nH: 0.43 (-74.36)	sH: 0.27 (-74.83)	eC: 0.42 (-74.4)			
		PR-nH: 0.11 (-75.7)						
	NS1	*PR-nH: 0.48 (-74.62);	nH: 0.52 (-74.55);	sH-Ja: 0.42 (-74.75);	eC-Ba: 0.5 (-74.59);	*eC-wC-Ba: 0.81 (-74.1)	7	12 (1)
		PR-nH-sH: 0.16 (-75.73)	nH-eC: 0.44 (-74.71)	sH: 0.32 (-75.02)	eC: 0.48 (-74.62)			
	TS2	PR-sH: 0.25 (-94.52);	*nH-eC: 0.95 (-93.18)	*sH-Ja: 0.97 (93.16)	*eC-Ba: 0.96 (-92.86)	*eC-wC-Ba: 0.95 (-92.86)	11	9
		PR-nH-sH-eC: 0.22 (-94.6);						
		PR-sH-eC: 0.22 (-94.6)						
BioGeoBEARS DEC model	NS1-j	*PR-nH-sH: 0.92	*nH: 0.68;	sH: 0.49;	eC: 0.49;	Ba: 0.4;	1	7 (6)
			eC: 0.29	Ja: 0.45	Ba: 0.48	eC: 0.2;		
						wC: 0.2		
	NS1	*PR-nH-sH: 0.73;	*nH-eC: 0.52;	*sH-Ja: 0.72	*eC-Ba: 0.84	*eC-wC-Ba: 0.66;	10	3
		PR-sH: 0.22	eC: 0.16			eC-Ba: 0.16;		
						wC-Ba: 0.16		
	TS1-j	*PR-sH: 0.72;	nH: 0.57;	*sH: 0.99	*eC: 0.81;	eC: 0.38;	1	9 (6)
		PR-nH-sH: 0.12	eC: 0.37		wC: 0.18	wC: 0.28;		
						eC-wC: 0.27		
	TS1	*PR-sH: 0.84	*nH-eC: 0.66;	*sH: 0.99	*eC: 0.65;	eC-wC-Ba: 0.45;	6	9 (5)
			nH: 0.3		wC: 0.21	eC-Ba: 0.43		

We excluded from the comparison the TS1 from Lagrange because of the unrealistic scenarios that were recovered (see text). Critical nodes for testing the Caribbean paleogeographical (vicariance) model are shown with their correspondent reconstructed ancestral geographical range. Preferred node distributions are highlighted in bold text and preceded by an asterisk (*). The number of well-supported vicariance and dispersal events were only counted when the relative probability of the best inference is two times larger than the following reconstructed distribution, in both immediate ancestral and daughter nodes. Dispersal events include the number of anagenetic range-switching and cladogenetic founder-events (the latter in parenthesis).

The estimation of the parameter *j* (founder-event speciation) significantly improved the DEC models. The global likelihood of TS1 was improved using BioGeoBEARS because, in contrast to Lagrange C++, we were able to constrain the area-connectivity through time slices. NS1-*j* preferred dispersal in critical nodes, i.e. the colonization of Jamaica, Cuba and the Bahamas, as well as a widespread origin of *Calisto* (PR-nH-sH) followed by vicariance. However, from all four models used in BioGeoBEARS, Akaike weights and likelihood-ratio test (LRT) suggested that TS1-*j* had a higher probability of being the best model, followed by TS1. Dispersal to Jamaica and the Bahamas are fully recovered in both TS1 and TS1-*j* from BioGeoBEARS, whereas vicariance is favoured as an explanation of the origin of Cuban *Calisto* only in TS1 analysis.

### Diversification processes within *Calisto*

The ΔAIC_RC_ critical value for small phylogenies, as estimated in *laser*, is 4 [47]. The observed value for *Calisto* is significantly higher than this threshold (ΔAIC_RC_ = 13), favouring a rate-variable diversification model. However, there was no statistical difference between the rate-variable models Yule-3-rates (Y3r) and the logistic density-dependent (DDL) (ΔAIC_Y3r – DDL_ = 3.8). The diversification of the main *Calisto* tree excluding the Cuban lineage also fits the rate-variable process (ΔAIC_RC_ = 11) better, but there was not strong preference among DDL, Yule-2, and −3-rates (ΔAIC_DDL – Y3r_ = 2.0; ΔAIC_DDL – Y2r_ = 3.8) (Table 5).

**Table 5:** **Diversification dynamics of** ***Calisto*** **as reconstructed by the R package** ***laser***

**Diversification rates for** ***Calisto***											
	**LH**	**AIC**	**r**_**1**_	**r**_**2**_	**r**_**3**_	**a**	**X**	**k**	**t_shift**_**1**_	**t_shift**_**2**_	**ΔAIC (yule-3-rates)**
pb	−32.301	66.602	0.07	-	-	-	-	-	-	-	13.32
bd	−32.301	68.602	0.07	-	-	0	-	-	-	-	15.32
ddx	−28.705	61.41	0.394	-	-	-	0.599	-	-	-	8.124
*ddl	**−26.579**	**57.158**	**0.175**	**-**	**-**	**-**	**-**	**40.119**	**-**	**-**	**3.871**
spvar	−28.84	63.681	0.229	-	-	0.004	-	-	-	-	10.39
exvar	−32.36	70.721	0.069	-	-	0.014	-	-	-	-	17.43
bothvar	−28.826	65.653	0.227	-	-	0.004	-	-	-	-	12.37
yule-2-rates	−26.056	58.112	0.094	0.014	-	-	-	-	4.079	-	4.826
*yule-3-rates	**−21.643**	**53.286**	**0.21**	**0.083**	**0.007**	**-**	**-**	**-**	**20.903**	**4.058**	**0**
**Diversification rates for** ***Calisto*** **excluding Cuban clade**											
	**LH**	**AIC**	**r**_**1**_	**r**_**2**_	**r**_**3**_	**a**	**X**	**k**	**t_shift**_**1**_	**t_shift**_**2**_	**ΔAIC (ddl)**
pb	−33.893	69.787	0.056	-	-	-	-	-	-	-	11.19
bd	−33.893	71.787	0.056	-	-	0	-	-	-	-	13.19
ddx	−29.428	62.855	0.55	-	-	-	0.889	-	-	-	4.258
*ddl	**−27.299**	**58.597**	**0.186**	**-**	**-**	**-**	**-**	**24.646**	**-**	**-**	**0**
spvar	−29.332	64.664	0.253	-	-	0.004	-	-	-	-	6.066
exvar	−33.951	73.902	0.055	-	-	0.018	-	-	-	-	15.3
bothvar	−29.323	66.646	0.251	-	-	0.004	-	-	-	-	8.048
*yule-2-rates	**−28.178**	**62.355**	**0.073**	**0.016**	**-**	**-**	**-**	**-**	**5.11**	**-**	**3.758**
*yule-3-rates	**−25.313**	**60.626**	**0.21**	**0.055**	**0.008**	**-**	**-**	**-**	**20.903**	**5.079**	**2.029**

The best models for our data is either DDL or yule-3-rates, which are highlighted in bold text and with an asterisk (*). Both models, nonetheless, predict a decreasing in diversification rates through time. Excluding the Cuban clade resulted in DDL, yule-2 or yule-3-rates as the main processes for the diversification of Hispaniolan lineages. LH: the best recovered log-likelihood, *r*_*i*_: net diversification rate at time *i* (λ_i_ - μ_i_), *a*: extinction fraction (μ_i_ / λ_i_), *X*: parameter controlling the magnitude of rates (only in DDX), *k*: parameter analogous to species “carrying capacity” (only in DDL), t_shift_i_: diversification shift at time *i*. The diversification models are pure-birth (pb), birth-death (bd), density-dependent speciation rate model following exponential (ddx) or logistic variants (ddl), exponential decline of speciation with constant extinction (spvar), exponential increase of extinction with constant speciation (exvar), speciation and extinction changes through time (bothvar), and pure birth models with *n* shifts in speciation (yule-n-rate).

From all rate-variable models in *DDD*, only those with a whole *Calisto* shift under diversity-dependent process are preferred with Akaike weights higher than 0.1. One single shift in the *K* parameter (“clade-level carrying capacity”) at 14 Ma fits 2–3 times better than shifts in *K* along with speciation or extinction rates. The decoupling of parameters for the Cuban taxa alone from the main *Calisto* tree was not enough to explain the radiation of the genus. Cuban and Hispaniolan taxa analyzed separately did not have constant diversification rates; rates changed possibly due to increased speciation, diversity-dependence processes, or a combination of both (Akaike Weights were unable to discern among models). Including the number of missing taxa into the models when possible did not affect the recovered estimations (Table 6).

**Table 6:** **Diversification dynamics of** ***Calisto*** **as reconstructed by the R package** ***DDD***

											**Analyses account 8 missing species**								
		**λ**_**0**_	**μ**_**0**_	**K**_**0**_	**λ**_**1**_	**μ**_**1**_	**K**_**1**_	**t_shift**	**log-LH**	**Akaike weight**	**λ**_**0**_	**μ**_**0**_	**K**_**0**_	**λ**_**1**_	**μ**_**1**_	**K**_**1**_	**t_shift**	**log-LH**	**Akaike weight**
	***Calisto*** **diversification**																		
CR0	constant λ and μ (birth-death)	0.07	0	-	-	-	-	-	−124.437	0.00	0.08	0	-	-	-	-	-	−123.853	0.00
CR1	λ declining as div-dep. No μ (DDL)	0.175	-	40.119	-	-	-	-	−118.715	0.03	-	-	-	-	-	-	-	-	-
CR2	div-dep with μ (DDL + E) depend in λ	0.163	0	43.055	-	-	-	-	−118.926	0.01	0.163	0	54.745	-	-	-	-	−118.774	0.02
SR0	Yule-2-rate	0.122	-	36.641	0.3	-	K_0_	13.567	−116.243	0.12	0.122	-	45.321	0.298	-	K1	13.567	−116.487	0.16
*SR1	**shift in K**	**0.306**	**0**	**13.132**	**λ**_**0**_	**μ**_**0**_	**36.62**	**13.567**	**−113.877**	**0.48**	**0.295**	**0**	**14.456**	**λ**_**0**_	**μ**_**0**_	**45.466**	**13.567**	**−114.384**	**0.47**
SR2	shift in K and μ	0.306	0	13.13	λ_0_	0	36.617	13.567	−113.878	0.18	0.3	0.006	14.342	λ_0_	0	45.355	13.567	−114.369	0.18
SR3	shift in K and λ	0.322	0	13.025	0.298	μ_0_	36.713	13.567	−113.863	0.18	0.289	0	14.507	0.298	μ_0_	45.414	13.567	−114.382	0.17
KI1	shift in K in subclade	0.133	0	27.172	λ_0_	μ_0_	Inf.	13.567	−118.634	0.00	0.139	0	37.103	λ_0_	μ_0_	Inf.	13.567	−121.355	0.00
KI2	shift in K and μ in subclade	0.133	0	27.159	λ_0_	0	Inf.	13.567	−118.651	0.00	0.139	0	36.965	λ_0_	0	Inf.	13.567	−121.371	0.00
KI3	shift in K and λ in subclade	0.164	0	25.591	0.115	μ_0_	Inf.	13.567	−118.319	0.00	0.17	0	34.419	0.114	μ_0_	Inf.	13.567	−120.853	0.00
KI4	shift in K, λ and μ in subclade	0.162	0	25.779	0.111	0	Inf.	13.567	−118.302	0.00	0.17	0	34.434	0.114	0	Inf.	13.567	−120.852	0.00
	**Hispaniolan lineages diversification**																		
CR0	constant λ and μ (birth-death)	0.056	0	-	-	-	-	-	−85.5	0.00	0.066	0	-	-	-	-	-	−84.936	0.00
CR1	λ declining as div-dep. No μ (DDL)	0.186	-	24.646	-	-	-	-	−78.905	0.19	-	-	-	-	-	-	-	-	-
CR2	div-dep with μ (DDL + E) depend in λ	0.165	0	26.665	-	-	-	-	−80.231	0.02	0.166	0	36.387	-	-	-	-	−79.968	0.04
SR0	Yule-2-rate	0.141	-	24	0.335	-	K_0_	12.843	−77.021	0.17	0.141	-	32	0.347	-	K1	12.843	−77.219	0.20
*SR1	**shift in K**	**0.33**	**0.001**	**11.991**	**λ**_**0**_	**μ**_**0**_	**23.918**	**14.352**	**−75.372**	**0.32**	**0.339**	**0.001**	**15.518**	**λ**_**0**_	**μ**_**0**_	**31.942**	**12.843**	**−75.477**	**0.42**
SR2	shift in K and μ	0.318	0.001	11.964	λ_0_	0	24	14.352	−75.219	0.14	0.327	0	15.593	λ_0_	0	32.023	12.844	−75.427	0.16
SR3	shift in K and λ	0.333	0	12.98	0.245	μ_0_	24.135	12.843	−75.09	0.16	0.295	0.001	15.743	0.355	μ_0_	31.948	12.843	−75.372	0.17
	**Cuban lineage diversification**																		
CR0	constant λ and μ (birth-death)	0.09	0	-	-	-	-	-	−40.903	0.01	-	-	-	-	-	-	-	-	-
CR1	λ declining as div-dep. No μ (DDL)	0.27	-	14.568	-	-	-	-	−38.31	0.14	-	-	-	-	-	-	-	-	-
CR2	div-dep with μ (DDL + E) depend in λ	0.219	0	17.498	-	-	-	-	−39.235	0.02	-	-	-	-	-	-	-	-	-
*SR0	**Yule-2-rate**	**0.099**	**-**	**14**	**0.493**	**-**	**K**_**0**_	**10.475**	**−35.409**	**0.35**	-	-	-	-	-	-	-	-	-
SR1	shift in K	0.502	0.012	2.323	λ_0_	μ_0_	13.644	11.894	−35.377	0.13	-	-	-	-	-	-	-	-	-
*SR2	**shift in K and μ**	**0.426**	**0.024**	**1.864**	**λ**_**0**_	**0**	**13.982**	**13.567**	**−33.981**	**0.20**	-	-	-	-	-	-	-	-	-
SR3	shift in K and λ	0.162	0.009	1.884	0.466	μ_0_	13.718	13.573	−34.287	0.15	-	-	-	-	-	-	-	-	-

The best models for each type of analyses, which include extinction and diversity-dependent processes, are highlighted in bold text and with an asterisk (*). λ is speciation rate, μ is extinction rate, K is species “carrying capacity” or a parameter analogous to it only in DDL. The estimated parameters to the right were calculated accounting missing taxa (8 species). The DDL model is not able to incorporate missing taxa whereas the Cuban clade in this study included all described species. A shift in K is recovered as the best explanation for the diversification patterns of *Calisto* and the Hispaniolan lineages. Yule-2-rate or a shift in K and μ are the best models to explain the diversification of the Cuban clade alone.

## Discussion

### Colonization of the Greater Antilles by *Calisto*

The variability in our dataset (39% and 28% of all characters were variable and phylogenetically informative respectively; Table 1) is similar to previous inter-generic studies in Nymphalidae [32,48,49], but relatively higher than intra-generic studies [50,51]. The genus *Calisto* is most likely a “relict” satyrine group that might have colonized the Greater Antilles during the uplift of GAARlandia (~35-33 Ma) [32]. Our dating estimates, indeed, confirm that it is an old and independent lineage, and its crown age (31 ± 5 Ma) provides evidence in support of the GAARlandia origin. Previous attempts to date the diversification of *Calisto* were done based only on a pairwise substitution rate for mitochondrial evolution [27]. This latter study deduced younger ages (4–8 Ma) but did not actually carry out a timing of the divergence analysis, rather they only calculated pairwise genetic distances with Kimura 2-parameter without an adequate model testing.

It is not the first time that GAARlandia is invoked to explain butterfly geographic range expansion. It is the case for the nymphaline subtribe Phyciodina [52], the satyrine subtribe Pronophilina [32], and certain lineages within the papilionid tribe Troidini [53]. The idea of indirect over-water dispersal by “hitch hiking” on hurricanes or flotsams rafts seems unlikely. Adult butterflies respond to incoming bad weather by taking refuge [36] whereas a high mortality of eggs, larvae and pupae is observed when they are exposed to marine water [54]. *Calisto*, when compared to most other butterflies, are rather sedentary, and hence the direct and indirect dispersal capabilities of *Calisto* make a dispersalist model less likely.

According to Iturralde-Vinent’s vicariance model [6], after GAARlandia, Hispaniola and Puerto Rico split around 20–30 Ma, whereas in our study, extant *Calisto* species in both islands have their most recent common ancestor at 27 ± 5 Ma. Furthermore, the Cuban clade branched off from a Hispaniola lineage at 21 ± 4 Ma, but did not apparently diversify into any extant *Calisto* until 14 ± 3 Ma, while the last aerial connection between blocks of Hispaniola and Cuba existed until 14–17 Ma. Therefore, the evolution of *Calisto* is better explained by the main predictions of the Caribbean paleogeographical model of colonization rather than the stochastic dispersalist scenario.

The inclusion of Jamaica into the vicariance model is less supported by the paleogeographical reconstructions, although a remote connection between the Blue Mountains block with GAARlandia has not been discarded [4,6]. The sole extant Jamaican *Calisto* split from its Hispaniolan sister taxa at 14 ± 4 Ma. At that time, large portions of Jamaica began to uplift and the entire island remained above water afterwards, and hence the colonization of Jamaica by rare long-distance dispersal events is the most likely explanation for the origin of the endemic sole species found there, *Calisto zangis*.

### Historical biogeography of *Calisto*

Biogeographical reconstructions were significantly improved when we constrained dispersal probability and area-connectivity following the paleogeographic history of the Caribbean. Moreover, we found for the first time, statistical support for long-distance dispersal in the colonization of the Bahamas and Jamaica by estimating a founder-event parameter using a more general DEC model. Vicariance was recovered as the main explanation for the first diversification event of *Calisto*, although we did not find a fully supported dispersal/vicariance origin for the Cuban clade. Whereas NS1 and TS1 in BioGeoBEARS and TS2 did significantly recover vicariance, other analyses did not favour either dispersal nor vicariance. This could be due primarily to, first, the assumptions made by the models and, second, the different approaches to node reconstruction. In the first case, vicariance is favoured when incorporating area connectivity through time (TS) but dispersal is recovered by adding the parameter *j* (founder-event or long-distance dispersal speciation). In the second case, Lagrange infers ancestral states by local optimization whereas BioGeoBEARS reports ancestral states under the most likely model. This difference is evidenced in NS1 (analysis replicated using both software programs) where vicariance is only reconstructed under the global most-likely inference by BioGeoBEARS.

We believe, given the paleogeographic scenario and our dating estimations which correlate with the former, that the most plausible explanation for the colonization of Cuba is vicariance. Furthermore, the whole of extant Cuban diversity is monophyletic and sister to a Hispaniola lineage, as predicted by the vicariant model. Dispersal into Cuba 10–25 Ma, on the other hand, was not “long-distance” because both land blocks were quite close apart, if not physically connected. Thus, if dispersal were actually the main process, we would expect several independent Cuban lineages of varied ages surviving to the present (see extinction rates in “Diversification of *Calisto*” section) (Figure 2).

Vicariance driving speciation within islands is significantly recovered for Hispaniolan fauna during two instances, at 10–13 Ma and 4–6 Ma. The first vicariant instance is independently evidenced in two lineages with simultaneous shifts in ancestral ranges, the *lyceius* and the *confusa-hysius* clades. The dating estimates are congruent with the major uplift of the Cordillera Central which might have provided new ecological opportunities and created isolated populations [39,40]. Presence of local adaptations are evidenced not only by the disjunctive distributions of several sister-species pairs found on the northern/southern Hispaniola paleoislands respectively (e.g. *C. tasajera* and *C. schwartzi* are allopatrically adapted to mesophilic and forested montane habitats in Cordillera Central (nH) and Sierra de Bahoruco (sH) [46,55,56]), but also by ecological niche restrictions. For instance, sister species-pairs within both major clades feed, as larvae, exclusively on distinct bunch grasses, and have morphologically adapted to specific altitudinal ranges. Species inhabiting lower altitude and warmer areas are smaller than their sister montane species [27,46], suggesting an adaptation for thermoregulatory efficiency [57].

The second instance of vicariant process within Hispaniola occurred during the Pliocene as evidenced in the *lyceius* and *chrysaoros* clades. Although an uplift of the Cordillera Central might have played a role in separating populations, the most likely explanation for the northern/southern paleoislands distributions might be related to the inundation of the Cul-de-Sac/Enriquillo depression, which acted as an effective barrier. Ecological niche shifts might be another plausible explanation for the *lyceus* clade members having differentiated during the Pliocene. As larvae, they feed on the bunchgrass *Uniola virgata*, which provides a unique niche and would have required significant adaptations [56].

The crown node ancestral distribution of Cuban and Bahamian *Calisto* is recovered as “eastern Cuba (eC)”. Its sister taxa are Hispaniolan lineages that occur in the northwestern Cordillera Central (Massif du Nord in Haiti) [22,46], which is the closest region to eastern Cuba. Dispersal to central and western Cuba from “eC” appears to be the likeliest biogeographic scenario [28], although vicariance as the main process is only detected in NS1 from BioGeoBEARS. Dispersal dates are in line with the closure of the Havana-Matanzas Channel at 8–5 Ma, as well as with the accretion of Bahamian shallows and keys in the Pliocene/Pleistocene [6]. The two Bahamian lineages have distinct ancestral areas, while *C. sibylla* has an older origin and its source area is “eC”, *C. apollinis* dispersed more recently from “western Cuba (wC)” (Figure 3).

### *Calisto* diversification on the Greater Antilles and the Bahamas

The species richness of *Calisto* across islands is largely unequal. Such a pattern has been previously reported as the consequence of island size and age, ecological limits and habitat diversity [8,38,58]. Munroe [59,60] pointed out that extant *Calisto* diversity is distributed unequally among islands more likely due to speciation rather than to differential immigration, and that extinction was extremely low, especially in Hispaniola. The calculation of diversification rates and ancestral states in this study suggested that the extant geographical distribution of *Calisto* reflects the rapid diversification within Hispaniola and Cuba during two instances, at 25 and 14 Ma, while inter-island flow was negligible for the entire genus.

*Calisto* is the most species-rich butterfly genus in the West Indies because it was able to expand its ecological niche (e.g. feeding on distinct bunchgrasses, tolerance to montane temperate and tropical conditions), which raised up the “ecological limits” on *Calisto* diversification. One sole change in the *K* parameter (“carrying-capacity for species diversity”) is enough to explain the evolution of the whole genus. The recovered date of this shift at 14 Ma is congruent with an increase in ecological opportunity in Hispaniola and Cuba and a time at which new environments were being created as a result of geological processes (e.g. uplift of Cordilleras, unification of Cuban land blocks) [39,40]. The decoupling of clade “carrying capacity” and/or diversification rates of the Cuban lineage as the only explanation for the genus species richness is not supported. Nonetheless, the arrival of *Calisto* to an unoccupied island of Cuba did certainly provide for new heretofore empty niches to be colonized. The most likely scenario for such a decoupling was at 14 Ma, as recovered in DR1 analysis. However, because such a date is confounded with the availability of new niches in Hispaniola, a model including one single shift in *K* for the whole genus was preferred.

Adaptive radiation and the origin of island endemism of West Indies insects remain statistically untested. Under a phylogenetic framework, indirect evidence of adaptive radiation could be inferred based on diversification rate shifts: i.e. a rapid increase followed by a gradual reduction of diversification rate under a diversity-dependent process [61]. *Calisto* butterflies might have undergone two increases in diversification before they rapidly reached a “carrying-capacity” limit. The first one occurred during the uplift of Cordillera Central at 25 Ma (SR1 analysis), triggering a growth in *Calisto* diversification rate until all available niches were gradually occupied, at which time, probably, the speciation rate declined linearly with diversity (*K* = 12). It is unlikely that the extinction rate rose, as it was near zero in all of our estimations. The second major radiation took place at 14 Ma (discussed above), but it is more plausible that the diversification rate increased due to a shift in *K* rather than by a sole increase in speciation rate. Furthermore, the “Inf.” values of *K* recovered for the Cuban clade in DR analyses might be an indication that the diversification rate has not yet reached its “ecological limit”. The Cuban clade, when analyzed independently, better fits a 2-yule-rate, with 5 times larger speciation rate at 10 Ma than when the lineage branched off at 21 Ma.

An intriguing question is why the observed diversification dynamics of *Calisto* on Cuba and Hispaniola were not replicated on Jamaica and Puerto Rico, the third and fourth largest islands of the West Indies, respectively. Whereas *Calisto* are usually locally adapted to particular habitats within Cuba and Hispaniola, the single species on each of the other two islands are widespread. While some diverse Hispaniolan lineages feed as larvae on bunch grasses, the Puerto Rican *C. nubila* is adapted to widespread-wide-blade grass feeding [56]. According to Turner, similar, relatively adaptable oviposition behaviour is exhibited by *C. zangis* of Jamaica [62]. Perhaps in this indiscriminate behaviour lies the explanation for the fact that these two species were able to colonize their entire respective islands instead of forming separate disjunctive populations as did their Hispaniolan congeners. Such wide distribution and relatively good dispersal abilities of these relatively larger *Calisto* species (Sourakov, pers. obs.) may have increased gene flow and hence prevented divergence. Further research on the natural history, dietary preferences and behaviour of *Calisto* is necessary to corroborate our speculations.

## Conclusions

The phylogenetic and biogeographical evidence presented in this study agrees with the Caribbean paleogeographical model of colonization (Figures 2 and 3). Vicariant models explaining the diversification of *Calisto* have already been proposed based on their extant geographical distribution [31,33,36,60], although some authors had favoured the alternative dispersalist explanation [27,63]. Here we observed that the evolution of *Calisto* passed through both vicariant processes and long-distance dispersals. However, the most important means for diversity origination in this largest genus of West Indies butterflies, was intra-island rapid radiation through key innovations (e.g. unusual larval hostplant, adaptation to montane, temperate and tropical conditions) and the availability of ecological niches triggered by environmental changes (e.g. accretion of mountain ranges, different island configuration and area-connectivity through time). Nonetheless, more rigorous tests and associations between ecological niche spectrum, phenotypic variability and selection within these butterflies are needed to give the adequate weight to abiotic factors (geographic and climatic) and niche specializations in the observed burst followed by a slowdown in diversification rates.

## Methods

### Taxon sampling

We included 36 out of the 44 described *Calisto* species (Additional file 2). Species sampling took place across the entire geographical distribution of the genus in the Greater Antilles, except for the Anegada Island where only one species occurs. Our analyses also included DNA sequences previously reported from taxa across the tribe Satyrini and *Calisto* [27-29,32] (Additional file 2). Species identifications were based on morphology and the DNA barcode region was used for further corroboration [64]. Voucher photographs are available at the Nymphalidae Systematics Group (NSG) Voucher Database (nymphalidae.utu.fi) and in BOLD (boldsystems.org).

### Dataset acquisition

Genomic DNA was isolated from two butterfly legs using the QIAGEN’s DNeasy kit. We used sequences of six standard molecular markers for nymphalid butterflies, one mitochondrial – COI (1487 bp) – and five nuclear genes – CAD (850 bp), EF-1α (1240 bp), GAPDH (691 bp), RpS5 (617) and *wingless* (400 bp). Primer pair sequences and laboratory protocols are described in [65]. DNA Sanger sequencing was carried out by the company Macrogen and each gene sequence was edited and manually aligned using the program BioEdit v7.0.5 [66]. Datasets were generated in different input formats using the web application VoSeq v1.7.0 [67].

### Phylogenetic analyses

We used single-gene and combined datasets. We partitioned our single-gene datasets by codon position and our combined dataset by gene sequences in all analyses. In addition we used character groupings of similar relative evolutionary rates as an alternative strategy for Bayesian Inference (BI) [68], after determining that the gene trees were not in conflict with each other. We used the software TIGER [69] to subdivide our combined dataset into 12 “bins” each containing a number of characters with similar relative rates: bin1 = 2739, bin2 to bin5 = 0, bin6 = 12, bin7 = 7, bin8 = 21, bin9 = 87, bin10 = 525, bin11 = 1269 and bin12 = 637. We combined the “bins” that contained fewer than 500 sites (bin2 to bin9) with the invariable bin1, resulting in four character groupings which were used for our alternative partitioning approach (Table 1).

We performed 1000 Maximum Likelihood (ML) pseudo-replicates analyses using RaXML v7.3.1 [70] on the Bioportal server [71], selecting the thorough bootstrap algorithm and the mix option for the evolutionary model. The BI analyses were carried out using MrBayes v3.2.1 [72] on the Bioportal server. We performed 10 million generations with sampling every 1000 generation and four chains, one cold and three heated, for two independent runs. The parameters and models of evolution were unlinked across character partitions. We selected the mixed evolutionary model option in all BI analyses whereas in the alternative partitioning strategy, we selected the corresponding model for each “bin” as calculated in jModelTest 0.1.1 [41] based on Bayesian Information Criterion (BIC). The convergence of the two runs on each BI was ascertained by visual inspection of the log-likelihoods stationary distribution, discarding the first 25% sampled trees, as well as by checking that the final average standard deviation of split frequencies was below 0.05 and that the potential scale reduction factor (PSRF) for each parameter was close to 1.

### Time of diversification estimates

Because there is no fossil record reported for the genus, we reconstructed a broader phylogeny including most of the representatives of the Satyrini subtribes that are closely related to *Calisto* [32] (Additional file 2) and constrained it with secondary calibration points from a fossil-calibrated Nymphalidae phylogeny [73]. We selected only one terminal per *Calisto* species to maximize the gene coverage in the resulting dataset. We also made an analysis excluding the genus *Euptychia* because long branch attraction affecting the position of *Calisto* has been reported [32]. The selected calibration points were chosen from well-supported monophyletic groups: the root of the tree to 49.1 ± 5 Ma, the crown age of the tribe Satyrina to 24.7 ± 4 Ma and the crown age of Euptychiina excluding *Euptychia*, *Paramacera* and *Cyllopsis* to 35.1 ± 4 Ma.

The dating analyses were run in BEAST v1.7.4 [74] and executed on the Bioportal server. We partitioned our dataset by gene sequence and set the corresponding substitution model as calculated in jModelTest (Table 1) and the uncorrelated log-normal relaxed clock model for each partition. We applied either the Birth-Death or the Calibrated Yule speciation processes as the tree prior in separate analyses to investigate the impact of this parameter on the final age estimates. In addition, the calibration points were modelled as either normal distributions (soft bounds) or uniform ranges (hard bounds). Finally, we set the mean rate of the molecular clock (ucld prior) with a uniform distribution between 0.0 and 10.0 and left other priors as default.

Each analysis was run four independent times for 50 million generations each and sampling trees and parameters every 5000th generation. We discarded the first 2500 sampled trees from each run as burnin. We verified in Tracer v1.5 the convergence and good mixing of MCMC as well as the Effective Sample Size of each estimated parameter to be higher than 200. Output .log and .tre files were combined in LogCombiner v1.7.4 after resampling a third of the post-burnin trees from each run. Trees were summarized in TreeAnnotator v1.7.4 into a single maximum clade credibility tree with node information calculated as mean heights.

### Historical biogeography reconstruction

We used our dated chronogram for *Calisto* as the input tree, excluding outgroups and *C. pulchella* because its distribution has been altered by sugar cane introduction, on which it is currently a pest [33]. The following subdivision of areas was set: “PR” – Puerto Rico; “nH” – the northern Hispaniola paleoisland, including Cordillera Central/Massif du Nord, Sierra de Neiba/Chaîne des Matheux, and eastern Hispaniola; “sH” – the southern Hispaniola paleoisland, including Sierra de Bahoruco/Chaîne de la Selle and Massif de la Hotte in Tiburón Peninsula; “Ja” – Jamaica; “eC” – the eastern Cuba, including Nipe – Sagua – Baraoca and Sierra Maestra mountain ranges; “wC” – the central and western Cuba, including Guamuhaya and Guaniguanico mountain ranges; “Ba” – the Bahamas. Distributional ranges of *Calisto* were taken from several sources [22,27-29,33,46].

We used the Dispersal-Extinction-Cladogenesis (DEC) model as implemented in Lagrange C++ [75,76]. DEC is a realistic and flexible model for biogeographical reconstructions that estimates the probabilities (likelihoods) of ancestral geographical distributions, and it allows the parameterization of dispersal through time according to the geological history of a region. We conducted analyses using a non-time-stratified approach (NS) and different dispersal rates across time slices (stratified, TS). The non-time-stratified analysis NS0 was conducted under default settings. The maximum distributional range was constrained to three areas and we excluded distributions with unlikely area-connectivity (e.g. Puerto Rico and Western Cuba) in NS1 analysis. TS1 used four time slices, subdividing the phylogeny at 5 Ma, 10 Ma and 20 Ma. Dispersal rate matrices were constructed according to the paleogeographical configuration on each time slice. Probabilities to disperse were set to 0.75 when two areas were adjacent, to 0.5 when two areas were weakly separated by a geographical barrier (e.g. the Cul-de-Sac/Enriquillo depression), to 0.1 when two areas were separated by water of a distance less than 200 km (e.g. northern Hispaniola and eastern Cuba), to 0.01 for long-distance dispersal, including one extra area and/or >200 km water-crossing (e.g. Puerto Rico to southern Hispaniola), and to 0.0001 for other kinds of long-distance dispersal.

We found a particular node in TS1 analysis to be unlikely (the Cuban-Bahamian subclade including *C. sibylla* and *C. apollinis*). This group had a crown age of 10 ± 2 Ma and an ancestral range eC-Ba after TS1. Paleogeographically, this is improbable because the Bahamas were submerged at least until the Pliocene (~5 Ma). We thus constrained such node to “eC” in TS2 because Lagrange, as it is currently implemented, does not allow the exclusion of unlikely area-connectivity through time slices.

Moreover, several sets of area distribution were independently constrained at the root of the *Calisto* tree to maximize the global likelihood of NS1 and TS2 and to compare the statistical support of likely ancestral ranges. We also used the R package BioGeoBEARS [77,78] which implements the DEC model similar to Lagrange C++ but with the possibility of increasing the number of free parameters. We allowed the founder-event speciation parameter *j* to be estimated in NS1-*j* and TS1-*j* to evaluate the importance of long-distance dispersal across islands. Another advantage of BioGeoBEARS is that distinct area-connection through time is allowed, hence we created an area-connectivity matrix for each time slice in TS1 and TS1-*j* (Figure 3).

### Diversification of *Calisto*

We used the packages *laser* [79], *ape* [80] and *DDD* [61,81] in R [82] to investigate the mode of diversification of extant *Calisto* taxa. Lineage Through Time (LTT) plots with confidence intervals representing a pure-birth null hypothesis model were made using *ape*. We compared different models of cladogenesis allowing temporal shifts in diversification rates using the Akaike Information Criterion differentials (ΔAIC_RC_ = AIC_RC_ (best rate-constant model) - AIC_RV_ (best rate-variable model)) as implemented in *laser*. We also computed the ΔAIC_RC_ separately for the *Calisto* phylogeny, excluding the Cuban species.

We used the R package *DDD* to fit the best phylogenetic diversification model that would explain the evolutionary history of *Calisto*. The analyses included three main models: a constant-rate evolution (CR), a shift in net diversification rate at some point in time (SR) and a decoupling of rates between the Cuban clade and the remaining taxa (DR). CR models incorporated either constant birth-death process (CR0), a decrease in speciation rate following a density-dependent process without extinction (CR1) or a decrease in speciation rate following a diversity-dependent process, including the estimation of extinction rate (CR2). SR models were set up to: one shift in speciation rate (yule-2-rate model) (SR0), one shift in species carrying capacity *K* (SR1), one shift in *K* and extinction rate (SR2), or one shift in *K* and speciation rate (SR3). DR models described one single shift in *K* for the Cuban clade (DR1), one shift in *K* and extinction rate for the Cuban clade (DR2), one shift in *K* and speciation rate for the Cuban group (DR3), and one shift in *K*, speciation and extinction rates for Cuban taxa (DR4). Moreover, we conducted CR and SR analyses for the main *Calisto* tree, excluding the Cuban clade, and for the Cuban clade independently. Comparisons between different phylogenetic diversification models were done using Akaike weights.

## Availability of supporting data

The data sets supporting the results of this article are available in the TreeBASE repository, in http://purl.org/phylo/treebase/phylows/study/TB2:S16186?format=html [83].

## Additional files

Additional file 1:
**Additional phylogenetic trees of** ***Calisto.*** In order of appearance in the pdf file: gene trees using COI (page 1), CAD (page 2), EF1a (page 3), GAPDH (page 4), RpS5 (page 5) and wingless (page 6) single gene datasets in MrBayes. Each node displays posterior probabilities values. Calibrated *Calisto* trees as estimated using BEAST including the genus *Euptychia* and using priors on calibration points and speciation process respectively: Normal and Birth-Death (BD) (page 7), Normal and Yule (page 8), Uniform and BD (page 9), Uniform and Yule (page 10). Calibrated *Calisto* trees excluding the genus *Euptychia* and using: Normal and BD (page 11), Normal and Yule (page 12), Uniform and BD (page 13), Uniform and Yule (page 14). On the calibrated trees, posterior probabilities are displayed on each node, 95% confidence interval for the dating estimates are shown as bars on each node, two red stars on every tree show the calibration points that were used in the present study and the y-axis represents time in million years. Subtribes names are represented on the left of each clade. Additional file 2:
**List of specimens and voucher information used in the present study.** A. xls file displaying each sequenced gene are presented with their corresponding GenBank accession number, unless the sequence is not available in the database where a “X” is shown. The hyphen “-” indicates unsuccessful DNA sequencing for that particular gene.

## Abbreviations

Ma: Million years ago
AIC: Akaike information criterion
DEC: Dispersal-Extinction-Cladogenesis model
BI: Bayesian inference
ML: Maximum likelihood
