# Supplementary material for: Causes of endemic radiation in the Caribbean: evidence from the historical biogeography and diversification of the butterfly genus Calisto (Nymphalidae: Satyrinae: Satyrini)
Source: BMC Evol Biol. 2014 Sep 16;14:199. doi: 10.1186/s12862-014-0199-7 (PMC4172866; doi:10.1186/s12862-014-0199-7)

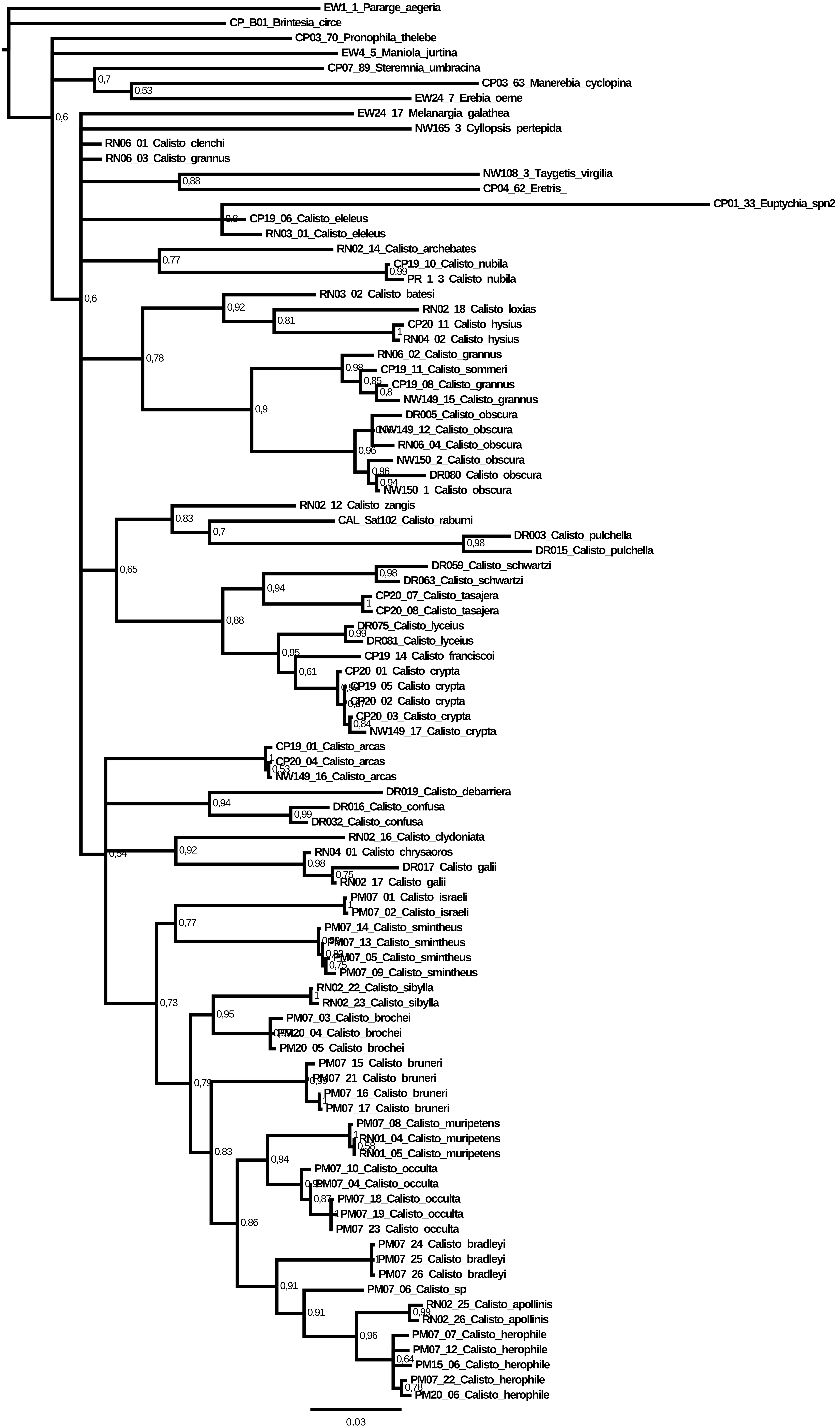
EW1_1_Pararge_aegeria
CP_B01_Brintesia_circe
CP03_70_Pronophila_thelebe
EW4_5_Maniola_jurtina
CP07_89_Steremnia_umbracina
CP03_63_Manerebia_cyclopina
EW24_7_Erebia_oeme
EW24_17_Melanargia_galathea
NW165_3_Cyllopsis_pertepida
RN06_01_Calisto_clenchi
RN06_03_Calisto_grannus
NW108_3_Taygetis_virgilia
CP04_62_Eretris_
CP01_33_Euptychia_spn2
CP19_06_Calisto_eleleus
RN03_01_Calisto_eleleus
RN02_14_Calisto_archebates
CP19_10_Calisto_nubila
PR_1_3_Calisto_nubila
RN03_02_Calisto_batesi
RN02_18_Calisto_loxias
CP20_11_Calisto_hysius
RN04_02_Calisto_hysius
RN06_02_Calisto_grannus
CP19_11_Calisto_sommeri
CP19_08_Calisto_grannus
NW149_15_Calisto_grannus
DR005_Calisto_obscura
NW149_12_Calisto_obscura
RN06_04_Calisto_obscura
NW150_2_Calisto_obscura
DR080_Calisto_obscura
NW150_1_Calisto_obscura
RN02_12_Calisto_zangis
CAL_Sat102_Calisto_raburni
DR003_Calisto_pulchella
DR015_Calisto_pulchella
DR059_Calisto_schwartzi
DR063_Calisto_schwartzi
CP20_07_Calisto_tasajera
CP20_08_Calisto_tasajera
DR075_Calisto_lyceius
DR081_Calisto_lyceius
CP19_14_Calisto_franciscoi
CP20_01_Calisto_crypta
CP19_05_Calisto_crypta
CP20_02_Calisto_crypta
CP20_03_Calisto_crypta
NW149_17_Calisto_crypta
CP19_01_Calisto_arcas
CP20_04_Calisto_arcas
NW149_16_Calisto_arcas
DR019_Calisto_debarriera
DR016_Calisto_confusa
DR032_Calisto_confusa
RN02_16_Calisto_clydoniata
RN04_01_Calisto_chrysaoros
DR017_Calisto_galii
RN02_17_Calisto_galii
PM07_01_Calisto_israeli
PM07_02_Calisto_israeli
PM07_14_Calisto_smintheus
PM07_13_Calisto_smintheus
PM07_05_Calisto_smintheus
PM07_09_Calisto_smintheus
RN02_22_Calisto_sibylla
RN02_23_Calisto_sibylla
PM07_03_Calisto_brochei
PM20_04_Calisto_brochei
PM20_05_Calisto_brochei
PM07_15_Calisto_bruneri
PM07_21_Calisto_bruneri
PM07_16_Calisto_bruneri
PM07_17_Calisto_bruneri
PM07_08_Calisto_muripetens
RN01_04_Calisto_muripetens
RN01_05_Calisto_muripetens
PM07_10_Calisto_occulta
PM07_04_Calisto_occulta
PM07_18_Calisto_occulta
PM07_19_Calisto_occulta
PM07_23_Calisto_occulta
PM07_24_Calisto_bradleyi
PM07_25_Calisto_bradleyi
PM07_26_Calisto_bradleyi
PM07_06_Calisto_sp
RN02_25_Calisto_apollinis
RN02_26_Calisto_apollinis
PM07_07_Calisto_herophile
PM07_12_Calisto_herophile
PM15_06_Calisto_herophile
PM07_22_Calisto_herophile
PM20_06_Calisto_herophile
0.03

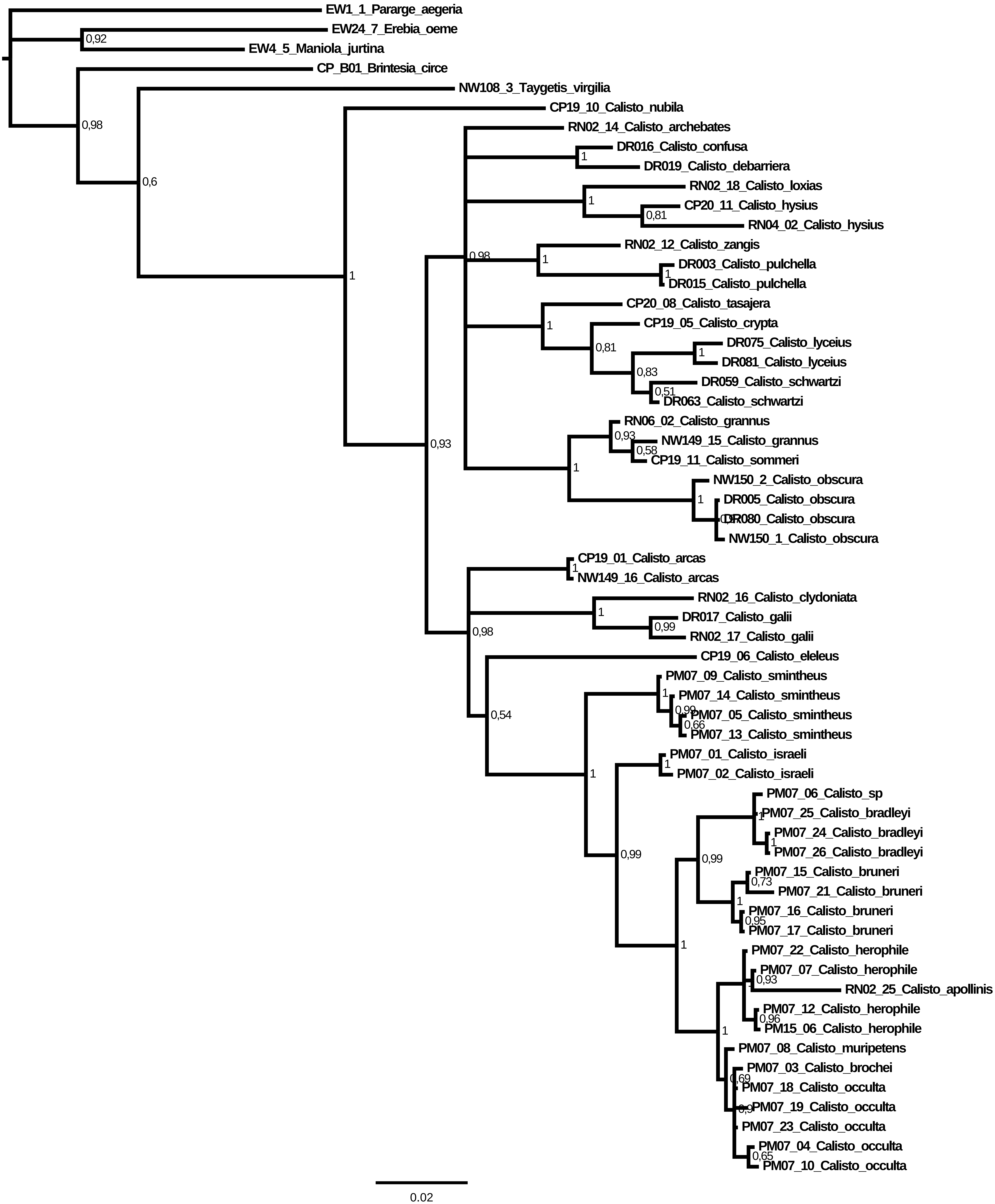
EW1_1_Pararge_aegeria
EW24_7_Erebia_oeme
0,92
EW4_5_Maniola_jurtina
CP_B01_Brintesia_circe
0,98
NW108_3_Taygetis_virgilia
0,6
CP19_10_Calisto_nubila
1
RN02_14_Calisto_archebates
DR016_Calisto_confusa
1
DR019_Calisto_debarriera
RN02_18_Calisto_loxias
1
CP20_11_Calisto_hysius
0,81
RN04_02_Calisto_hysius
RN02_12_Calisto_zangis
0,98
1
DR003_Calisto_pulchella
1
DR015_Calisto_pulchella
CP20_08_Calisto_tasajera
1
CP19_05_Calisto_crypta
0,81
DR075_Calisto_lyceius
1
DR081_Calisto_lyceius
0,83
DR059_Calisto_schwartzi
0,51
DR063_Calisto_schwartzi
0,93
RN06_02_Calisto_grannus
0,93
NW149_15_Calisto_grannus
0,58
CP19_11_Calisto_sommeri
1
NW150_2_Calisto_obscura
1
DR005_Calisto_obscura
DR080_Calisto_obscura
NW150_1_Calisto_obscura
CP19_01_Calisto_arcas
1
NW149_16_Calisto_arcas
RN02_16_Calisto_clydoniata
1
DR017_Calisto_galii
0,99
RN02_17_Calisto_galii
0,98
CP19_06_Calisto_eleleus
0,54
PM07_09_Calisto_smintheus
1
PM07_14_Calisto_smintheus
0,99
PM07_05_Calisto_smintheus
0,66
PM07_13_Calisto_smintheus
1
PM07_01_Calisto_israeli
1
PM07_02_Calisto_israeli
PM07_06_Calisto_sp
1
PM07_25_Calisto_bradleyi
PM07_24_Calisto_bradleyi
1
PM07_26_Calisto_bradleyi
0,99
0,99
PM07_15_Calisto_bruneri
0,73
PM07_21_Calisto_bruneri
1
PM07_16_Calisto_bruneri
0,95
PM07_17_Calisto_bruneri
1
PM07_22_Calisto_herophile
PM07_07_Calisto_herophile
0,93
RN02_25_Calisto_apollinis
PM07_12_Calisto_herophile
0,96
PM15_06_Calisto_herophile
1
PM07_08_Calisto_muripetens
PM07_03_Calisto_brochei
0,69
PM07_18_Calisto_occulta
0,9
PM07_19_Calisto_occulta
PM07_23_Calisto_occulta
PM07_04_Calisto_occulta
0,65
PM07_10_Calisto_occulta
0.02

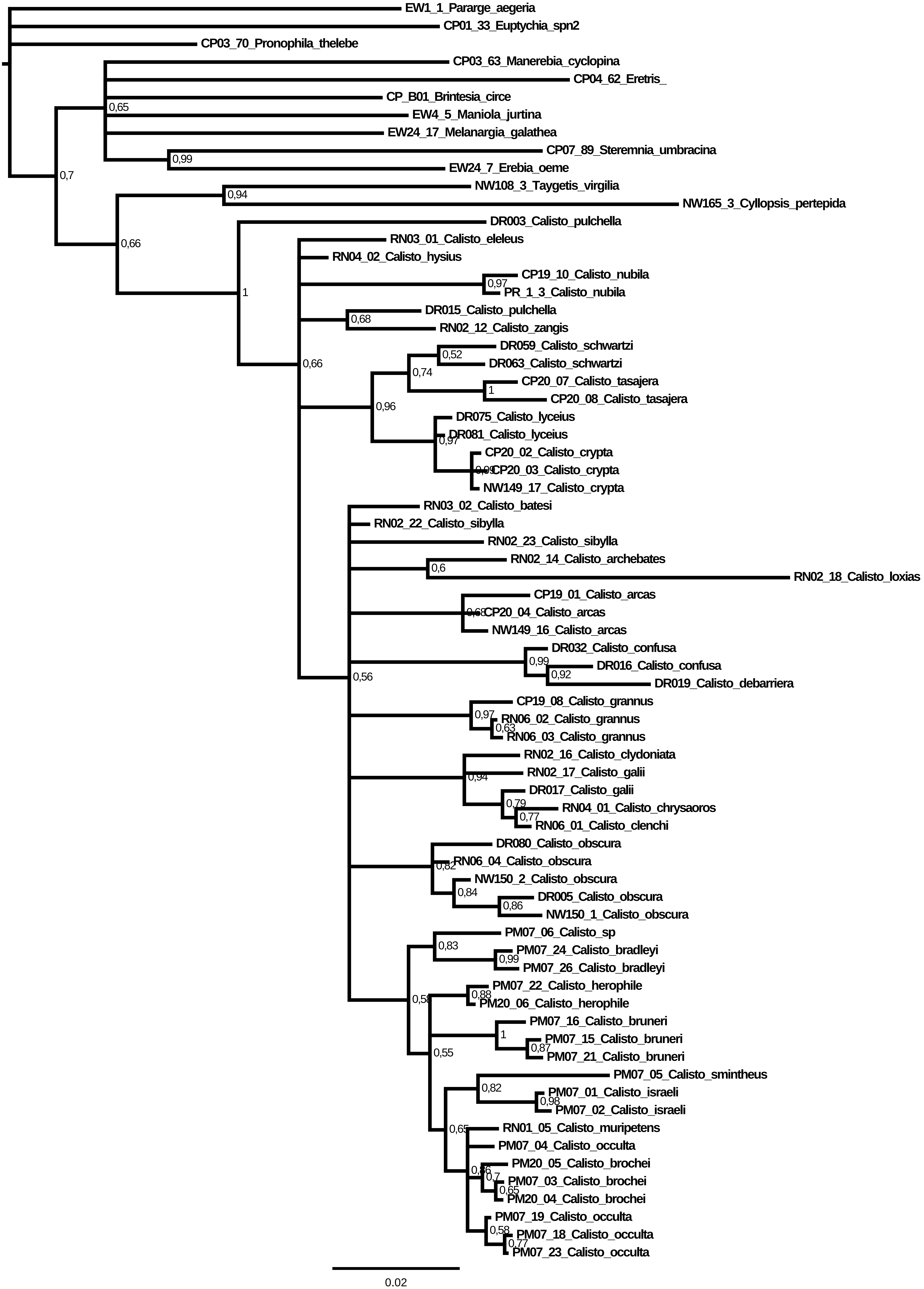
EW1_1_Pararge_aegeria
CP01_33_Euptychia_spn2
CP03_70_Pronophila_thelebe
CP03_63_Manerebia_cyclopina
CP04_62_Eretris_
CP_B01_Brintesia_circe
0,65
EW4_5_Maniola_jurtina
EW24_17_Melanargia_galathea
CP07_89_Steremnia_umbracina
0,99
EW24_7_Erebia_oeme
0,7
NW108_3_Taygetis_virgilia
0,94
NW165_3_Cyllopsis_pertepida
DR003_Calisto_pulchella
0,66
RN03_01_Calisto_eleleus
RN04_02_Calisto_hysius
CP19_10_Calisto_nubila
0,97
PR_1_3_Calisto_nubila
1
DR015_Calisto_pulchella
0,68
RN02_12_Calisto_zangis
DR059_Calisto_schwartzi
0,52
0,66
DR063_Calisto_schwartzi
0,74
CP20_07_Calisto_tasajera
1
CP20_08_Calisto_tasajera
0,96
DR075_Calisto_lyceius
DR081_Calisto_lyceius
0,97
CP20_02_Calisto_crypta
CP20_03_Calisto_crypta
NW149_17_Calisto_crypta
RN03_02_Calisto_batesi
RN02_22_Calisto_sibylla
RN02_23_Calisto_sibylla
RN02_14_Calisto_archebates
0,6
RN02_18_Calisto_loxias
CP19_01_Calisto_arcas
CP20_04_Calisto_arcas
NW149_16_Calisto_arcas
DR032_Calisto_confusa
0,99
DR016_Calisto_confusa
0,92
0,56
DR019_Calisto_debarriera
CP19_08_Calisto_grannus
0,97
RN06_02_Calisto_grannus
0,63
RN06_03_Calisto_grannus
RN02_16_Calisto_clydoniata
RN02_17_Calisto_galii
0,94
DR017_Calisto_galii
0,79
RN04_01_Calisto_chrysaoros
0,77
RN06_01_Calisto_clenchi
DR080_Calisto_obscura
RN06_04_Calisto_obscura
0,82
NW150_2_Calisto_obscura
0,84
DR005_Calisto_obscura
0,86
NW150_1_Calisto_obscura
PM07_06_Calisto_sp
0,83
PM07_24_Calisto_bradleyi
0,99
PM07_26_Calisto_bradleyi
PM07_22_Calisto_herophile
0,88
PM20_06_Calisto_herophile
PM07_16_Calisto_bruneri
1
PM07_15_Calisto_bruneri
0,87
0,55
PM07_21_Calisto_bruneri
PM07_05_Calisto_smintheus
0,82
PM07_01_Calisto_israeli
0,98
PM07_02_Calisto_israeli
RN01_05_Calisto_muripetens
PM07_04_Calisto_occulta
PM20_05_Calisto_brochei
0,7
PM07_03_Calisto_brochei
0,65
PM20_04_Calisto_brochei
PM07_19_Calisto_occulta
0,58
PM07_18_Calisto_occulta
0,77
PM07_23_Calisto_occulta
0.02

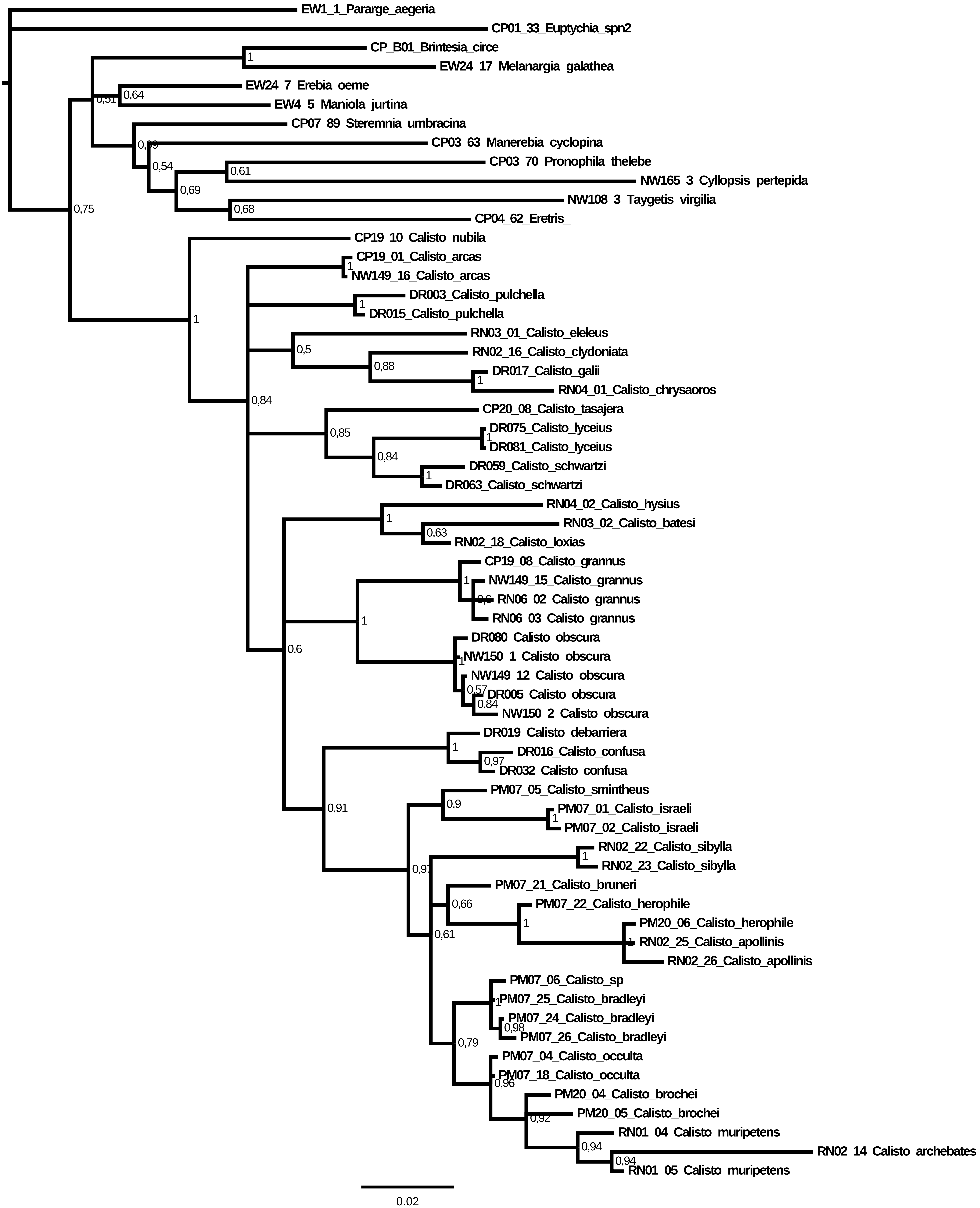
EW1_1_Pararge_aegeria
CP01_33_Euptychia_spn2
CP_B01_Brintesia_circe
1
EW24_17_Melanargia_galathea
EW24_7_Erebia_oeme
0,64
EW4_5_Maniola_jurtina
CP07_89_Steremnia_umbracina
CP03_63_Manerebia_cyclopina
0,54
CP03_70_Pronophila_thelebe
0,61
NW165_3_Cyllopsis_pertepida
0,69
NW108_3_Taygetis_virgilia
0,68
0,75
CP04_62_Eretris_
CP19_10_Calisto_nubila
CP19_01_Calisto_arcas
1
NW149_16_Calisto_arcas
DR003_Calisto_pulchella
1
1
DR015_Calisto_pulchella
RN03_01_Calisto_eleleus
0,5
RN02_16_Calisto_clydoniata
0,88
DR017_Calisto_galii
1
RN04_01_Calisto_chrysaoros
0,84
CP20_08_Calisto_tasajera
0,85
DR075_Calisto_lyceius
1
DR081_Calisto_lyceius
0,84
DR059_Calisto_schwartzi
1
DR063_Calisto_schwartzi
RN04_02_Calisto_hysius
1
RN03_02_Calisto_batesi
0,63
RN02_18_Calisto_loxias
CP19_08_Calisto_grannus
1
NW149_15_Calisto_grannus
RN06_02_Calisto_grannus
1
RN06_03_Calisto_grannus
DR080_Calisto_obscura
0,6
NW150_1_Calisto_obscura
1
NW149_12_Calisto_obscura
0,57
DR005_Calisto_obscura
0,84
NW150_2_Calisto_obscura
DR019_Calisto_debarriera
1
DR016_Calisto_confusa
0,97
DR032_Calisto_confusa
PM07_05_Calisto_smintheus
0,9
PM07_01_Calisto_israeli
0,91
1
PM07_02_Calisto_israeli
RN02_22_Calisto_sibylla
1
RN02_23_Calisto_sibylla
PM07_21_Calisto_bruneri
0,66
PM07_22_Calisto_herophile
1
PM20_06_Calisto_herophile
0,61
RN02_25_Calisto_apollinis
RN02_26_Calisto_apollinis
PM07_06_Calisto_sp
PM07_25_Calisto_bradleyi
1
PM07_24_Calisto_bradleyi
0,98
PM07_26_Calisto_bradleyi
0,79
PM07_04_Calisto_occulta
PM07_18_Calisto_occulta
0,96
PM20_04_Calisto_brochei
PM20_05_Calisto_brochei
0,92
RN01_04_Calisto_muripetens
0,94
RN02_14_Calisto_archebates
0,94
RN01_05_Calisto_muripetens
0.02

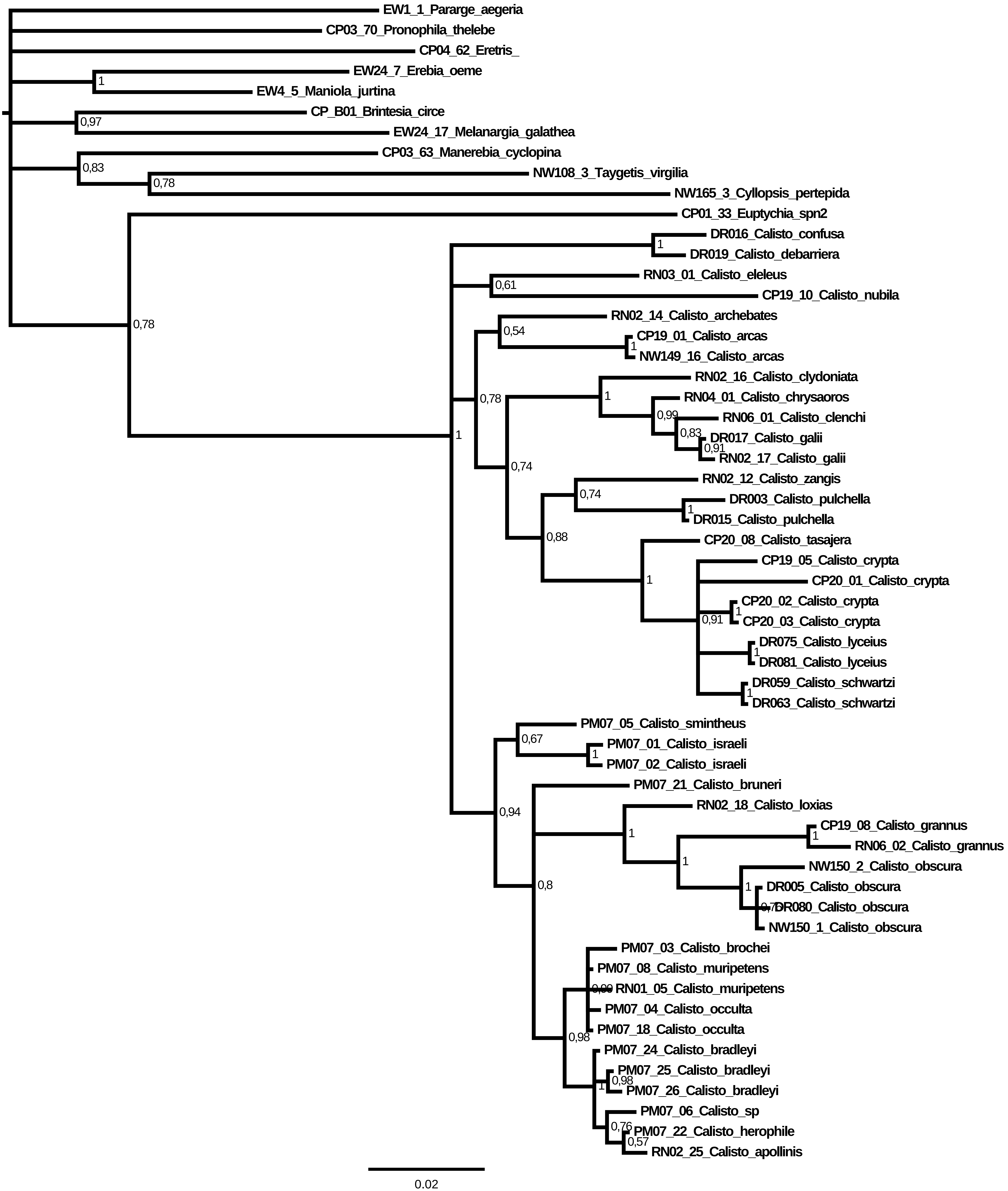

EW1_1_Pararge_aegeria
CP03_70_Pronophila_thelebe
CP04_62_Eretris_
EW24_7_Erebia_oeme
1
EW4_5_Maniola_jurtina
CP_B01_Brintesia_circe
0,97
EW24_17_Melanargia_galathea
CP03_63_Manerebia_cyclopina
0,83
NW108_3_Taygetis_virgilia
0,78
NW165_3_Cyllopsis_pertepida
CP01_33_Euptychia_spn2
0,78
DR016_Calisto_confusa
1
DR019_Calisto_debarriera
RN03_01_Calisto_eleleus
0,61
CP19_10_Calisto_nubila
RN02_14_Calisto_archebates
0,54
CP19_01_Calisto_arcas
1
NW149_16_Calisto_arcas
RN02_16_Calisto_clydoniata
0,78
1
RN04_01_Calisto_chrysaoros
0,99
RN06_01_Calisto_clenchi
0,83
DR017_Calisto_galii
0,91
RN02_17_Calisto_galii
1
0,74
RN02_12_Calisto_zangis
0,74
DR003_Calisto_pulchella
1
DR015_Calisto_pulchella
0,88
CP20_08_Calisto_tasajera
CP19_05_Calisto_crypta
1
CP20_01_Calisto_crypta
CP20_02_Calisto_crypta
1
0,91
CP20_03_Calisto_crypta
DR075_Calisto_lyceius
1
DR081_Calisto_lyceius
DR059_Calisto_schwartzi
1
DR063_Calisto_schwartzi
PM07_05_Calisto_smintheus
0,67
PM07_01_Calisto_israeli
1
PM07_02_Calisto_israeli
PM07_21_Calisto_bruneri
0,94
RN02_18_Calisto_loxias
CP19_08_Calisto_grannus
1
1
RN06_02_Calisto_grannus
1
NW150_2_Calisto_obscura
0,8
1
DR005_Calisto_obscura
DR080_Calisto_obscura
NW150_1_Calisto_obscura
PM07_03_Calisto_brochei
PM07_08_Calisto_muripetens
RN01_05_Calisto_muripetens
PM07_04_Calisto_occulta
PM07_18_Calisto_occulta
0,98
PM07_24_Calisto_bradleyi
PM07_25_Calisto_bradleyi
1
0,98
PM07_26_Calisto_bradleyi
PM07_06_Calisto_sp
0,76
PM07_22_Calisto_herophile
0,57
RN02_25_Calisto_apollinis
0.02

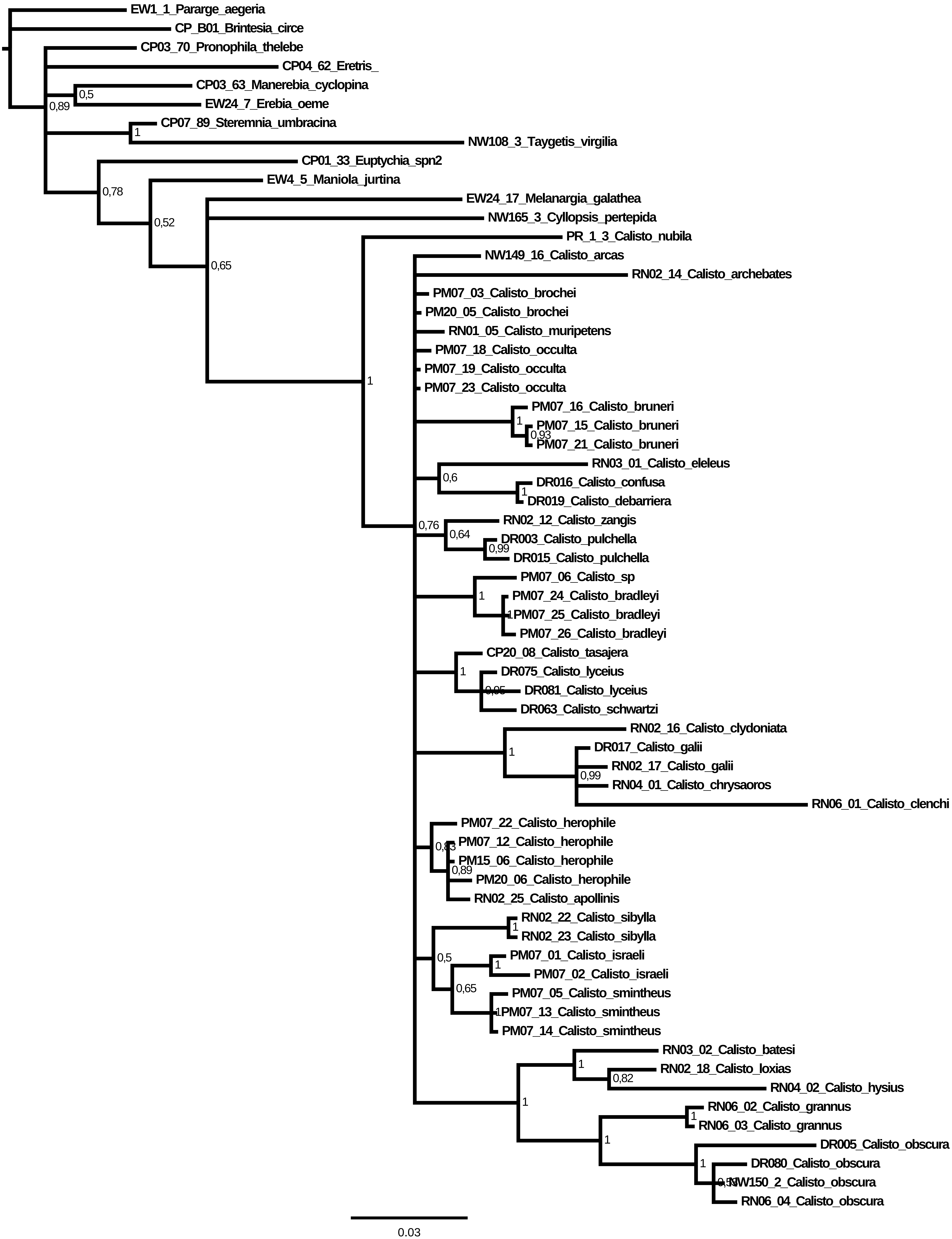
EW1_1_Pararge_aegeria
CP_B01_Brintesia_circe
CP03_70_Pronophila_thelebe
CP04_62_Eretris_
CP03_63_Manerebia_cyclopina
0,5
EW24_7_Erebia_oeme
0,89
CP07_89_Steremnia_umbracina
1
NW108_3_Taygetis_virgilia
CP01_33_Euptychia_spn2
EW4_5_Maniola_jurtina
0,78
EW24_17_Melanargia_galathea
0,52
NW165_3_Cyllopsis_pertepida
PR_1_3_Calisto_nubila
0,65
NW149_16_Calisto_arcas
RN02_14_Calisto_archebates
PM07_03_Calisto_brochei
PM20_05_Calisto_brochei
RN01_05_Calisto_muripetens
PM07_18_Calisto_occulta
PM07_19_Calisto_occulta
1
PM07_23_Calisto_occulta
PM07_16_Calisto_bruneri
1
PM07_15_Calisto_bruneri
0,93
PM07_21_Calisto_bruneri
RN03_01_Calisto_eleleus
0,6
DR016_Calisto_confusa
1
DR019_Calisto_debarriera
RN02_12_Calisto_zangis
0,76
0,64
DR003_Calisto_pulchella
0,99
DR015_Calisto_pulchella
PM07_06_Calisto_sp
PM07_24_Calisto_bradleyi
1
1
PM07_25_Calisto_bradleyi
PM07_26_Calisto_bradleyi
CP20_08_Calisto_tasajera
1
DR075_Calisto_lyceius
0,95
DR081_Calisto_lyceius
DR063_Calisto_schwartzi
RN02_16_Calisto_clydoniata
1
DR017_Calisto_galii
RN02_17_Calisto_galii
0,99
RN04_01_Calisto_chrysaoros
RN06_01_Calisto_clenchi
PM07_22_Calisto_herophile
PM07_12_Calisto_herophile
0,83
PM15_06_Calisto_herophile
0,89
PM20_06_Calisto_herophile
RN02_25_Calisto_apollinis
RN02_22_Calisto_sibylla
1
RN02_23_Calisto_sibylla
PM07_01_Calisto_israeli
0,5
1
PM07_02_Calisto_israeli
0,65
PM07_05_Calisto_smintheus
1
PM07_13_Calisto_smintheus
PM07_14_Calisto_smintheus
RN03_02_Calisto_batesi
1
RN02_18_Calisto_loxias
0,82
RN04_02_Calisto_hysius
1
RN06_02_Calisto_grannus
1
RN06_03_Calisto_grannus
1
DR005_Calisto_obscura
1
DR080_Calisto_obscura
0,53
NW150_2_Calisto_obscura
RN06_04_Calisto_obscura
0.03

Dated phylogeny: including the genus *Euptychia*, calibration points normally distributed and Birth-Death tree process

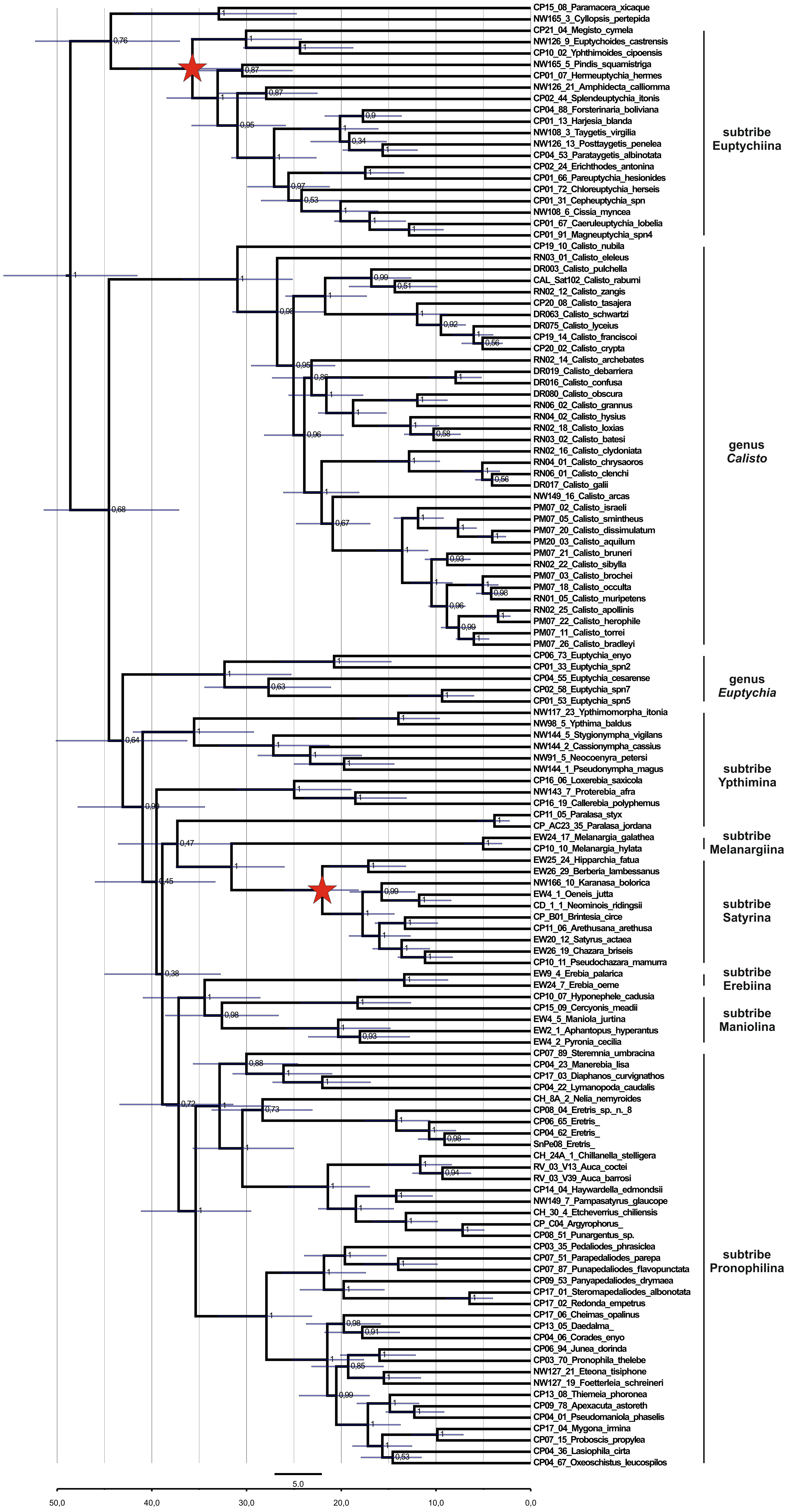

Dated phylogeny: including the genus *Euptychia*, calibration points normally distributed and Yule tree process

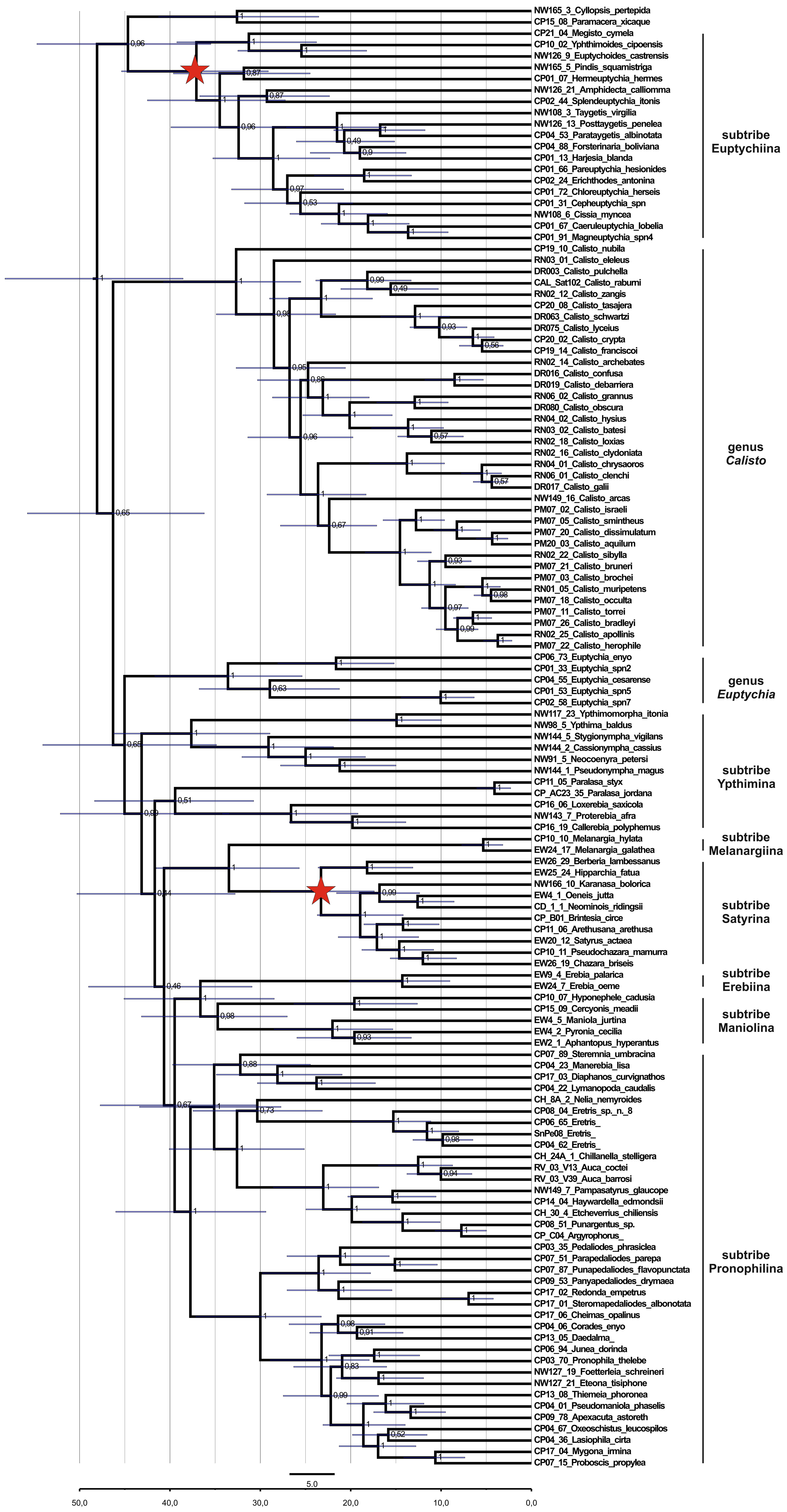

Dated phylogeny: including the genus *Euptychia*, calibration points uniformly distributed and Birth-Death tree process

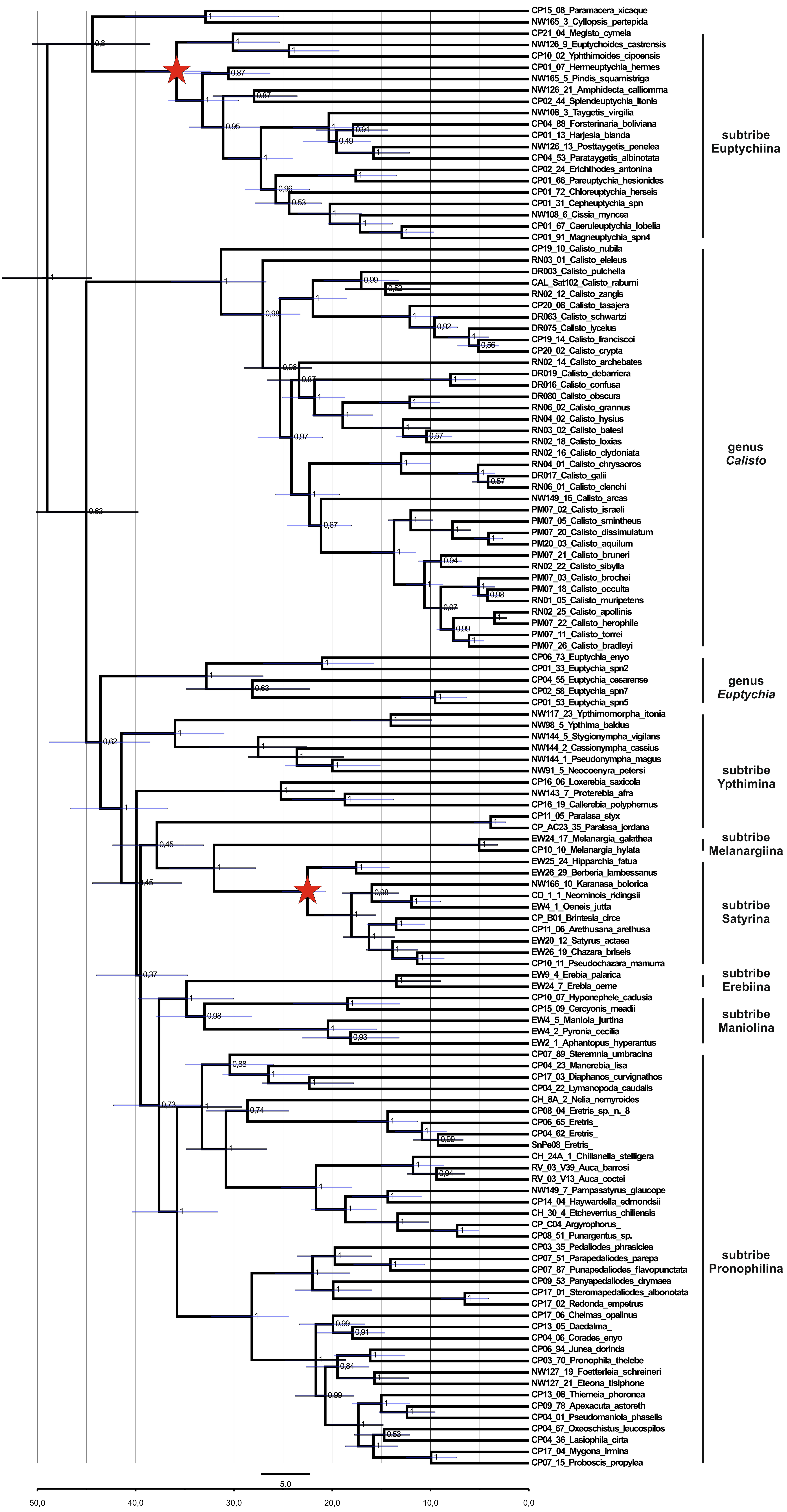

Dated phylogeny: including the genus *Euptychia*, calibration points uniformly distributed and Yule tree process

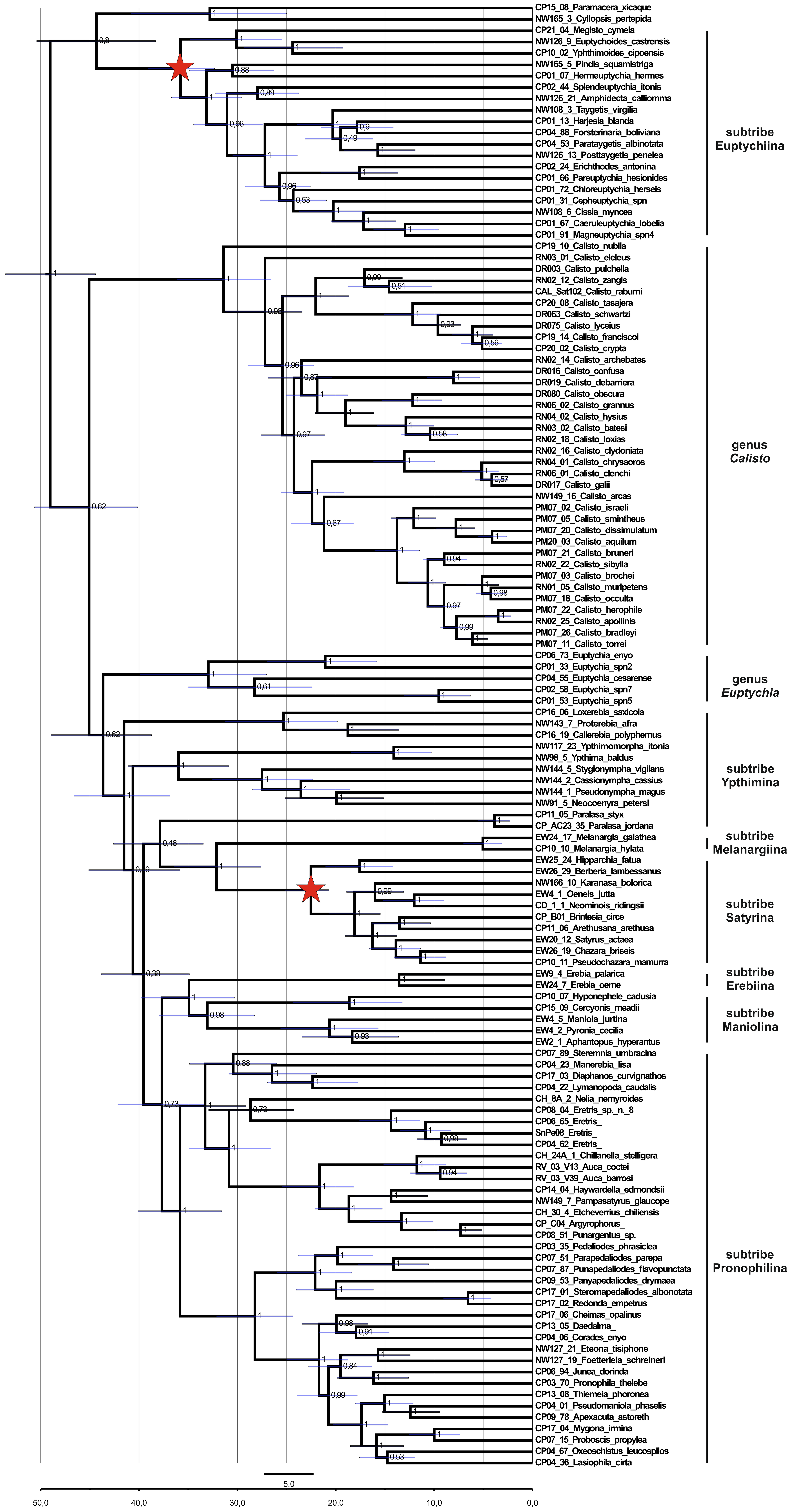

Dated phylogeny: excluding the genus *Euptychia*, calibration points normally distributed and Birth-Death tree process

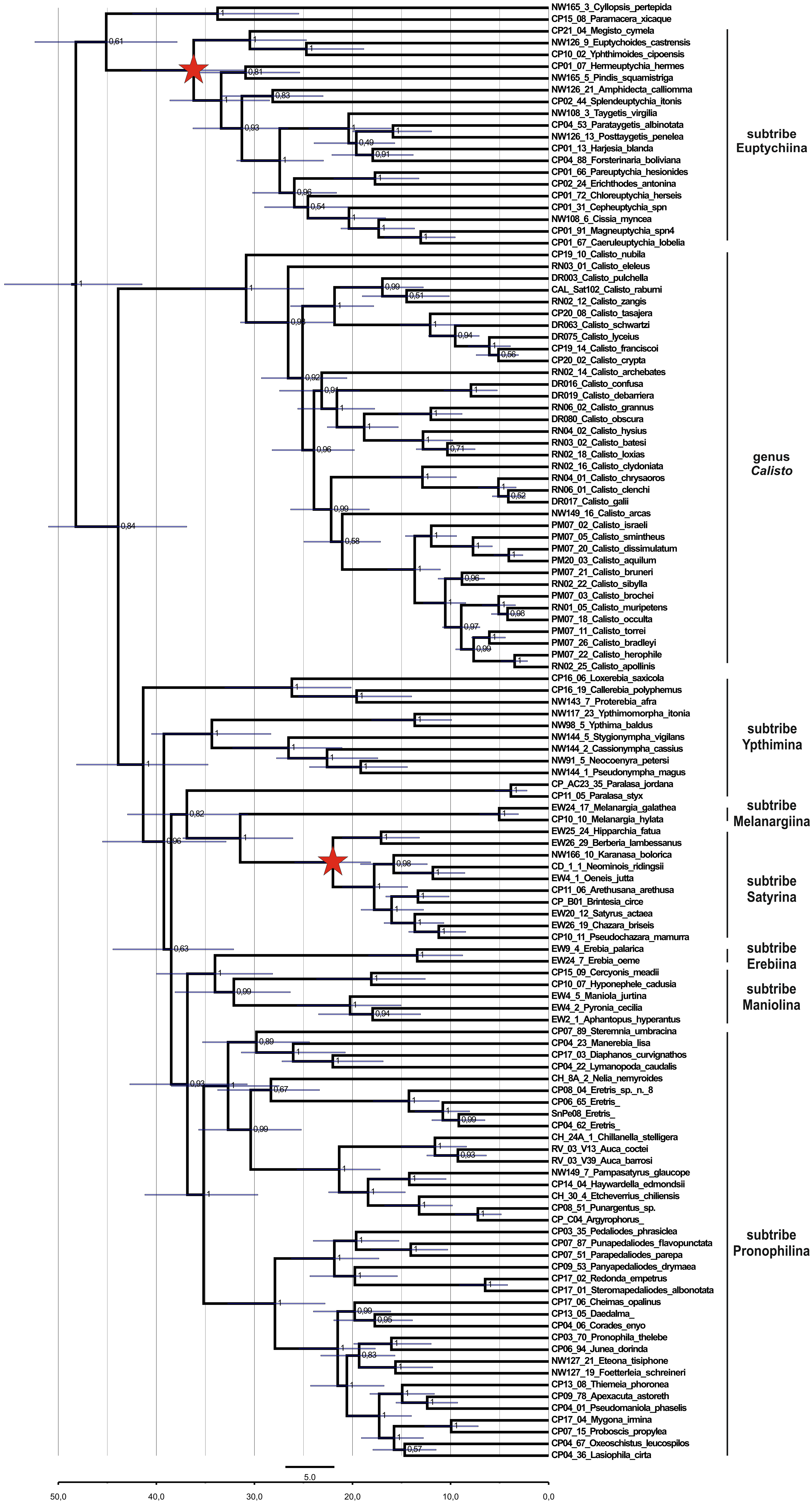

Dated phylogeny: excluding the genus *Euptychia*, calibration points normally distributed and Yule tree process

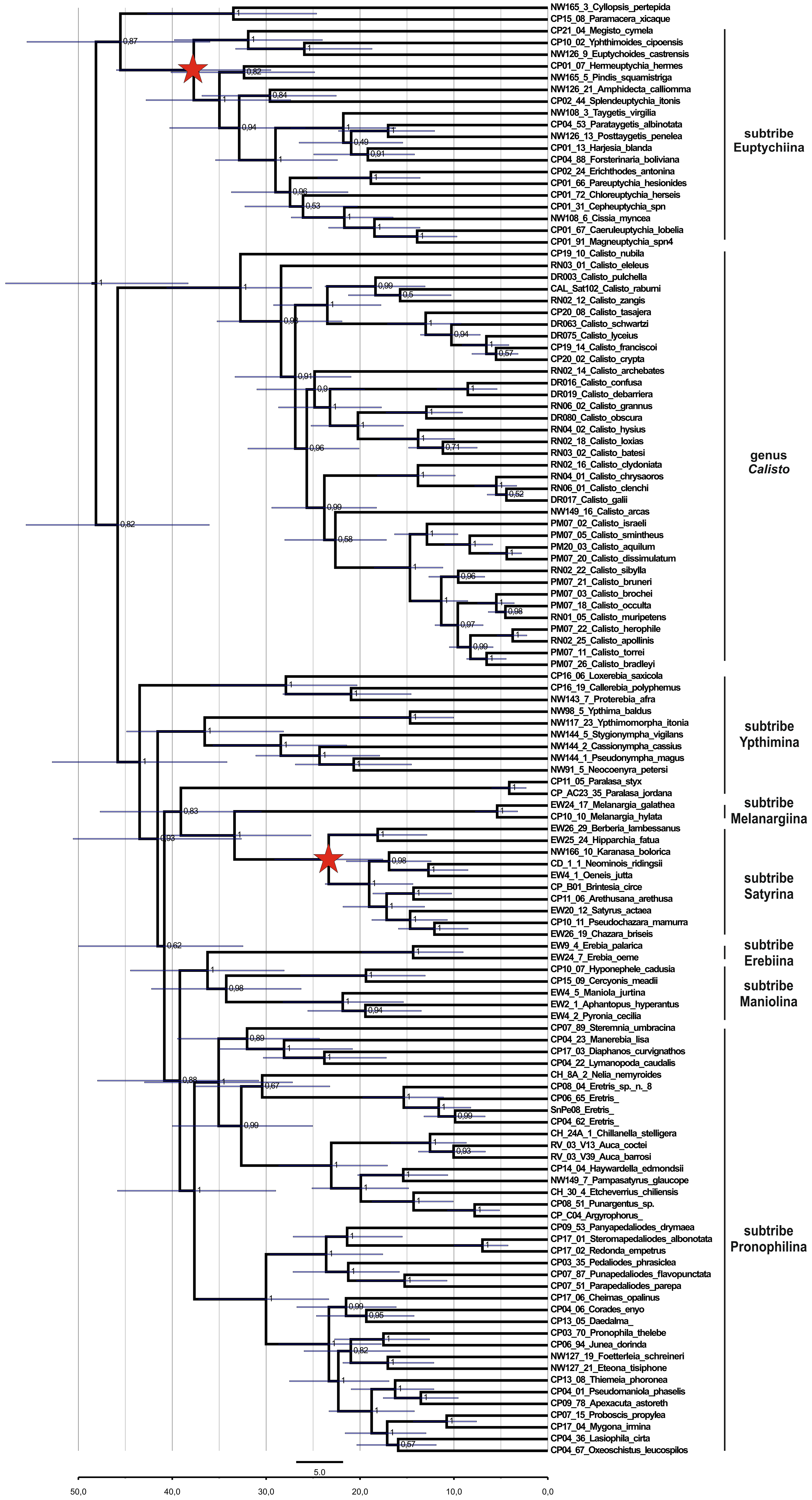

Dated phylogeny: excluding the genus *Euptychia*, calibration points uniformly distributed and Birth-Death tree process

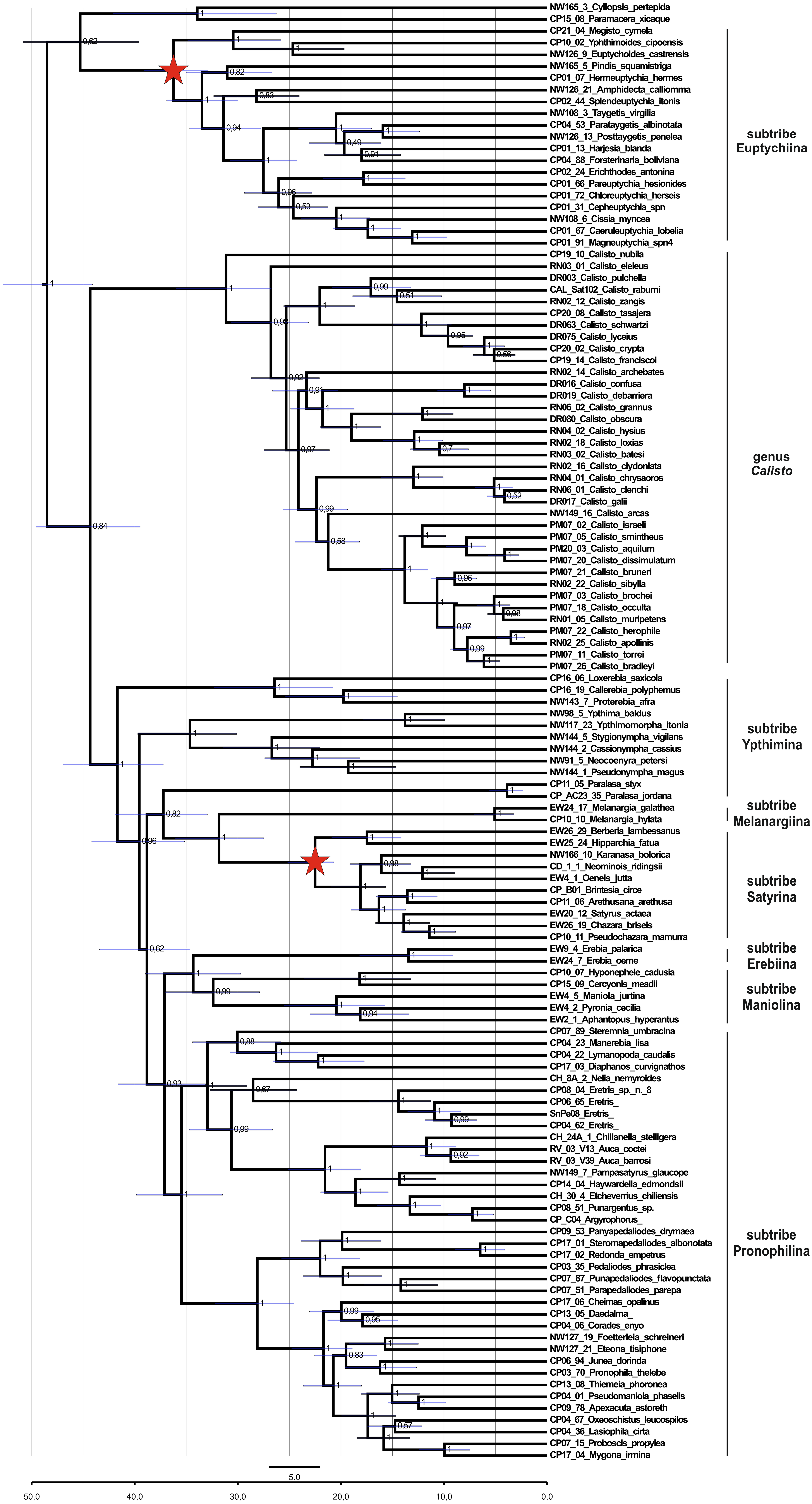

Dated phylogeny: excluding the genus *Euptychia*, calibration points uniformly distributed and Yule tree process

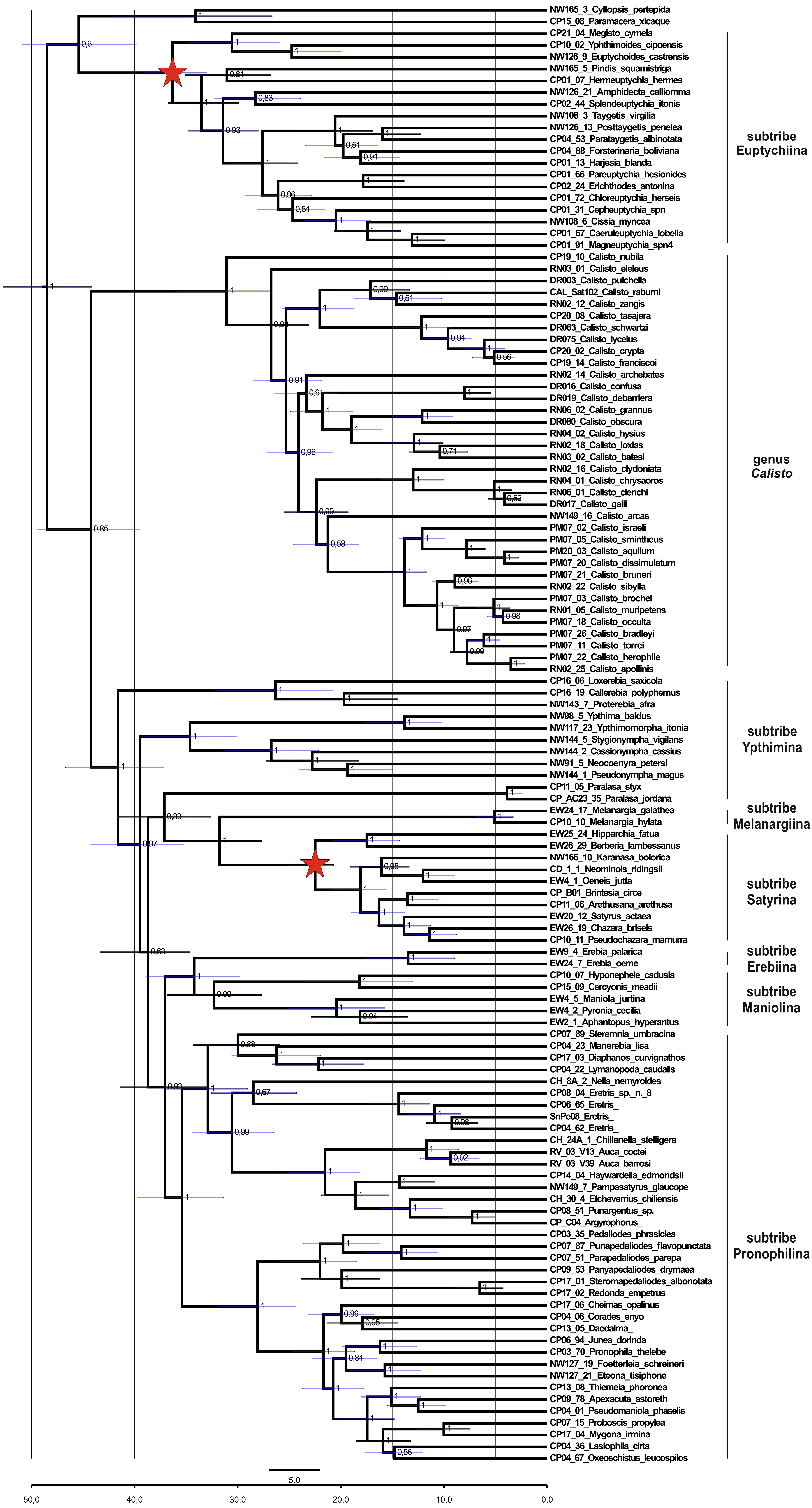

Supplement: Additional file 1: — Additional phylogenetic trees of Calisto. In order of appearance in the pdf file: gene trees using COI (page 1), CAD (page 2), EF1a (page 3), GAPDH (page 4), RpS5 (page 5) and wingless (page 6) single gene datasets in MrBayes. Each node displays posterior probabilities values. Calibrated Calisto trees as estimated using BEAST including the genus Euptychia and using priors on calibration points and speciation process respectively: Normal and Birth-Death (BD) (page 7), Normal and Yule (page 8), Uniform and BD (page 9), Uniform and Yule (page 10). Calibrated Calisto trees excluding the genus Euptychia and using: Normal and BD (page 11), Normal and Yule (page 12), Uniform and BD (page 13), Uniform and Yule (page 14). On the calibrated trees, posterior probabilities are displayed on each node, 95% confidence interval for the dating estimates are shown as bars on each node, two red stars on every tree show the calibration points that were used in the present study and the y-axis represents time in million years. Subtribes names are represented on the left of each clade. [file 12862_2014_199_MOESM1_ESM.pdf]
